# A Novel Prognostic Biomarker Panel for Early-Stage Colon Carcinoma

**DOI:** 10.3390/cancers13235909

**Published:** 2021-11-24

**Authors:** Pablo Azcue, David Guerrero Setas, Ignacio Encío, Berta Ibáñez-Beroiz, María Mercado, Ruth Vera, María Luisa Gómez-Dorronsoro

**Affiliations:** 1 Department of Health Science, Public University of Navarra, 31008 Pamplona, Spain; ignacio.encio@unavarra.es (I.E.); berta.ibanez.beroiz@navarra.es (B.I.-B.); 2 Department of Pathology, University Hospital of Navarra, 31008 Pamplona, Spain; dguerres@navarra.es (D.G.S.); mr.mercado.gutierrez@navarra.es (M.M.); 3 Campus Arrosadia, Public University of Navarra, 31006 Pamplona, Spain; 4 Molecular Pathology of Cancer Group–Navarrabiomed, 31008 Pamplona, Spain; 5 Department of Medical Oncology, University Hospital of Navarra, 31008 Pamplona, Spain; ruth.vera.garcia@navarra.es; 6 Institute for Health Research Navarra (IdISNA), 31008 Pamplona, Spain; 7 Unit of Methodology-Navarrabiomed-University Hospital of Navarra, 31008 Pamplona, Spain; 8 Research Network on Health Services Research and Chronic Diseases (REDISSEC), 31008 Pamplona, Spain

**Keywords:** PD-L1, GLUT-1, MUC2, e-cadherin, CDX2, CMS, colon cancer, prognostic biomarker

## Abstract

**Simple Summary:**

Decision about treatment choice in early-stage colon cancer may be difficult for clinicians because of a lack of published data. Colon cancer is a very heterogeneous disease, but some previous efforts have helped us elucidate potential biomarkers for characterizing patients. Our goal is to create a panel of biomarkers capable of differentiating patients with a low, medium, and high risk of death or relapse. Our results suggest that, by combining PD-L1, GLUT-1, and mismatch repair proteins in a biomarker panel, patients could be significantly and evenly divided into one of these three groups. The resulting biomarker panel has the potential clinical value that, by being able to classify a patient with early colon cancer as being at high risk of death or tumor evolution, they could benefit from a more aggressive early treatment, while this approach might not be needed for low-risk patients.

**Abstract:**

Molecular characterization of colorectal cancer has helped us understand better the biology of the disease. However, previous efforts have yet to provide significant clinical value in order to be integrated into clinical practice for patients with early-stage colon cancer (CC). The purpose of this study was to assess PD-L1, GLUT-1, e-cadherin, MUC2, CDX2, and microsatellite instability (dMMR) and to propose a risk-panel with prognostic capabilities. Biomarkers were immunohistochemically assessed through tissue microarrays in a cohort of 144 patients with stage II/III colon cancer. A biomarker panel consisting of PD-L1, GLUT-1, dMMR, and potentially CDX2 was constructed that divided patients into low, medium, and high risk of overall survival or disease-free survival (DFS) in equally sized groups. Compared with low-risk patients, medium-risk patients have almost twice the risk of death (HR = 2.10 (0.99–4.46), *p* = 0.054), while high-risk patients have almost four times the risk (HR = 3.79 (1.77–8.11), *p* = 0.001). The multivariate goodness of fit was 0.756 and was correlated with Kaplan–Meier curves (*p* = 0.002). Consistent results were found for DFS. This study provides a critical basis for the future development of an immunohistochemical assessment capable of discerning early-stage CC patients as a function of their prognosis. This tool may aid with treatment personalization in daily clinical practice and improve survival outcomes.

## 1. Introduction

Colorectal cancer (CRC) is the second most common cause of cancer deaths worldwide [1]. The overall 5-year patient survival rate has recently risen to almost two-thirds [2], partly due to curative oncological colon or rectal surgery, neoadjuvant treatments, and better long-term functional outcomes once the metastatic state has been reached [3].

CRC is a highly heterogeneous disease; earlier efforts have helped us understand the molecular events that play a crucial role in prognosis and treatment decisions. This is the case for microsatellite instability (MSI) or mismatch repair (MMR) protein deficiency for adjuvant therapy and KRAS (exon2), BRAF (V600E), and PIK3CA for anti-epidermal growth factor (anti-EGFR) therapy in the metastatic setting [4,5,6,7,8]. Additionally, technical advances have enabled further characterization through molecular profiling, including candidate cancer genes [9], Jass classification [10], Ogino classification [11], colorectal cancer intrinsic subtypes [12], and most recently, the consensus molecular subtype (CMS) [13].

It has become clearer that molecular events and gene alterations play a crucial role in the tumoral evolution. However, the previously mentioned characterization efforts have not been able to provide results of sufficient clinical significance to merit inclusion in international guidance and to change daily clinical practice. Nevertheless, these efforts have helped elucidate the main molecular signaling pathway aberrations and interactions, gene expression profiles, and pathological characteristics contributing to the complex tumoral evolution process [14,15].

The CMS classification has come closest to providing predictive capabilities with therapeutic implications through transcriptomics [16]. However, it continues not to be part of clinical practice, probably due to the highly specialized resources needed to carry it out, the failure to ascribe a defined molecular profile to 13% of the population, and the lack of a clear prognostic or predictive value (especially for the CMS2 and CMS3 subtypes). Nonetheless, the CMS classification has helped us better understand the molecular targets that can prove useful for biomarker selection. In a nutshell, the CMS2 or canonical subtype is characterized by chromosomal instability, with marked WNT and MYC signaling activation pathways with APC and TP53 mutations. The CMS3 or metabolic subtype is characterized by metabolic dysregulation with KRAS and APC mutations. CMS2 and CMS3 subtypes share characteristics with CMS1 (MSI-immune) and CMS4 (mesenchymal) subtypes, presenting a mixed prognosis in an early-stage and metastatic setting [17,18,19,20]. Further efforts are needed to find biomarkers capable of characterizing these patients, which are ideally easier to assess and implement in clinical practice.

Programmed cell death protein 1 (PD-1) and its ligand (PD-L1), glucose transporter 1 (GLUT-1), e-cadherin, mucin 2 (MUC2), and caudal-related homeobox transcription factor 2 (CDX2) are proteins that share plausible mechanisms with the CMS subtypes and their molecular profiles. They are also feasible to assess through immunohistochemistry (IHC), which might satisfy the need to clarify the prognosis of early-stage colon cancer patients.

Programmed cell death protein 1 (PD-1) or CD279 is an inhibitory receptor that is expressed by T cells during activation. PD-L1 expression in the tumor microenvironment interacts with PD-1 in tumor-infiltrating lymphocytes, attenuating the tumor-triggered T-cell response [21]. The activation of the WNT/β-catenin pathway by this receptor is among the signaling pathways that have been elucidated in CRC. It has been linked to progression [22] and the epithelial–mesenchymal transition (EMT) [23]. Additionally, PD-L1 receptor inhibition has been associated with the immune-related lymphocyte response that produces TGFβ [24]. Consequently, potential synergistic effects have recently been proposed between immune checkpoint inhibitors and TGFβ receptor inhibitors, with therapeutic implications in CRC [25,26].

Glucose transporter 1 (GLUT-1) is a transmembrane protein glucose transporter that is crucial in the glucose metabolism. Overexpression of GLUT-1 has been reported in different types of solid tumors including CRC [27,28], and it has been related to cancer cell proliferation and the provision of stress protection to the tumor [29]. GLUT-1 has been proposed as a potential marker for malignant transformation, with a probable direct link between the degree of overexpression and aggressiveness [30,31].

As a member of the calcium-dependent cell adhesion molecules (CAMs), e-cadherin is believed to be one of the most important adhesion molecules of epithelial tissues [32]. Downregulation of e-cadherin has been associated with tumor progression, loss of differentiation, and metastasis, probably due to its involvement in the EMT [33,34,35]. The value of e-cadherin as a potential biomarker for CRC is still controversial, although it has been associated with increasingly worse prognosis as the tumor stage advances [35,36,37]. Downregulation of e-cadherin modulates the WNT/β-catenin signaling pathway, which transcribes genes such as c-Myc and cyclin D1 [34,38].

MUC2 is a glycosylated protein that makes up part of the secreted gel-forming mucins [39]. It is normally expressed in the healthy epithelium of the colon but the alteration of MUC2 expression of tumor cells may play an important role in tumor progression. It has been suggested that the level of expression is lower in CRC [40,41,42], specifically in the early-stage setting [43,44]. Furthermore, low expression was significantly associated with lymph node metastasis, poor cellular differentiation, and advanced tumor stage in CRC [45], while retaining normal expression with improved outcomes [46]. Epithelial growth factor (EGF) and TGF-α seem to play a role in regulating MUC2 expression trough RAS kinases.

Homeobox protein CDX2 is expressed in early intestinal development and is involved in regulating the proliferation and differentiation of intestinal epithelial cells. It is expressed in the nuclei of epithelial cells in the intestinal tract [47]. CDX2 loss of expression has been primarily documented in poorly differentiated advanced intestinal carcinomas as a biomarker of poor prognosis [48,49,50,51]. Recent publications have reported the lack of expression of CDX2 as a predictive biomarker, with independence of MSI status [50,52,53].

Given the above, the objectives of the current analysis were to assess the abovementioned biomarkers on the basis of a molecular characterization of early-stage colon cancer patients and to propose a novel IHC biomarker panel with prognostic capabilities. The proposed panel should ideally be based on a robust and standardized assessment methodology, so that it may easily be implemented in hospitals and laboratories with immunohistochemical experience.

We hypothesize that the expression of PD-L1, GLUT-1, e-cadherin, MUC2, and CDX2, in addition to the MMR proteins in tumor samples, can help create an IHC biomarker panel that is able to discern patient prognosis in early-stage colon cancer.

## 2. Materials and Methods

This study follows up on previously published work [54]. It was performed in accordance with the World Medical Association Declaration of Helsinki and was approved by the Regional Clinical Research Ethics Committee (CEIC) Pyto2017/51 Cod. MOL_CRC, 15 May 2018. Patient consent was waived due to the use of stored tumor samples for research purposes, in compliance with current Spanish and European Union legislation (resolutions 1387/2017 (08/11) and 193/2018 (06/03) of the Navarra Health Service—Osasunbidea).

A cohort of 162 patients diagnosed with stage II/III CRC at the Hospital Complex of Navarra between 2009 and 2013 underwent surgery with curative intent, and samples were obtained. Tumors were classified according to the TNM Classification of Malignant Tumors, seventh edition [55]. Patients were followed up until 1 October 2018. Their data were anonymized and analyzed in the Department of Pathology. Patients with insufficient tumor material, who were lost to follow-up for more than 3 years, who had fewer than two evaluable IHC-stained blocks, who were missing baseline characteristics, or whose tumor was located rectally were excluded from the analysis.

A cohort of 144 patients with stage II/III colon carcinoma (CC) were included in the study for analysis. Tissue microarrays (TMA) were constructed, stained, revealed, and digitalized as previously described [54]. TMA sections were immunohistochemically stained against PD-L1, GLUT-1, e-cadherin, MUC2, CDX2, and MMR proteins.

The antibodies used for each biomarker were anti-PD-L1 (SP142; RTU; Roche, Tucson, AZ, USA), anti-GLUT-1 (Clone SMP498; 1:200; Thermo Fisher Scientific, Waltham, MA, USA), anti-e-cadherin (Clone 36; RTU; Roche, Tucson, AZ, USA), anti-MUC2 (Clone CCP58; 1:50; Dako, Glostrup, Denmark), and anti-CDX2 (Clone PA0535; RTU; Novocastra, Newcastle, UK). The MMR proteins used to determine MMR status were MLH1 (Clone PA0610; RTU; Biocare, Pacheco, CA, USA), MSH2 (Clone FE-11; 1:100; Calbiochem, San Diego, CA, USA), MSH6 (Clone PM265AA; RTU; Biocare, Pacheco, CA, USA), and PMS2 (Clone PM344AA; RTU; Biocare, Pacheco, CA, USA). Immunostaining was performed with Vision Leica Biosystems’ Bond-Max automatic immunostainer or Roche’s BenchMark XT Ventana automatic immunostainer with the OptiView DAB detection and amplification kit.

### 2.1. Immunohistochemical Assessment and Scoring

The most consistent methodology for IHC assessment and scoring for each antibody, in the current or a similar setting, was established from a review of the literature. Each array was assessed by one expert pathologist and one trained senior scientist. When found, discrepancies were evaluated and resolved by a third expert pathologist. All three evaluators were blinded to the patients’ clinical data and outcomes.

GLUT-1 is not usually expressed in normal colonic epithelium; however, when there is pathological expression, it is localized in the cytoplasm, mainly in the membrane [30,31,56,57,58]. Scoring is based on the percentage of expression in the cytoplasm or membrane as described in similar studies [31,59,60,61]. Absence of expression of GLUT-1 was scored as 0, expression in fewer than 30% of the cells of the sample was scored as 1, expression in 30% to fewer than 50% was scored of the cells of the sample as 2, expression in more than 50% but not more than 90% of the cells on the sample was scored as 3, and expression in more than 90% of the cells of the sample was scored as 4. GLUT-1 low expression was concluded for scores of 2 or more, and GLUT-1 high expression was ascribed for scores of 3 or more and was considered positive for statistical purposes.

E-cadherin is normally expressed in the membrane, and its loss of expression leads to it being assessed as abnormal [33,62]. Scoring is based on stain intensity (0–3) multiplied by the percentage of expression (0–4), rendering a final score between 0 and 12 [35,43]. Stain intensity was scored as absent (0), weak (1), moderate (2), and strong (3). The percentage expression was scored as complete absence (0), <10% (1), 10–50% (2), >50–80% (3), and >80% (4) of tumor cells. Loss of e-cadherin expression was concluded for final scores of ≤6 and was considered positive for statistical purposes.

MUC2 is normally expressed in the perinuclear cytoplasm of the goblet cells in normal colonic mucosa, but expression may vary in tumor cells [42,43,63,64]. The scoring is based on the percentage of expression in tumor cells [63,64,65,66]. Percentage expression was scored 0 for values of <25%, 1 for values between 25% and <50%, and 2 for values of ≥50% of the tumor cells. MUC2 was considered as retained expression for a score of 2 and loss of expression for a score of 0 or 1. Loss of expression was considered positive for statistical purposes.

CDX2 is expressed in the nucleus of normal colonic mucosa [67]. Scoring is based on the percentage of tumor cell expression, with a score of 0 for values of <50%, 1 for values between 50% and <95%, and 2 for values ≥ 95% [52,53,68,69]. CDX2 was considered as retained expression for scores of 2 and loss of expression for a score of 0 or 1. Loss of expression was considered positive for statistical purposes.

MMR proteins and PD-L1 expression were assessed in accordance with a previously published methodology [54].

A summarized methodology for assessment and scoring can be found in Table S1. Scanned images and examples of scoring assessment for each biomarker are presented in Figure S1.

### 2.2. Statistical Analysis

Baseline characteristics and study variable frequencies were summarized as percentages and means. MMR-deficient (dMMR) and MMR-proficient (pMMR) status groups were compared using the *t*-test or Mann–Whitney U test depending on the nature and normality of the variables and using the chi-squared test or Fisher’s exact test for categorical ones, depending on whether the expected number of cases was over five for all cells in the contingency table or not.

Overall survival (OS) was defined as time from surgery to death due to any cause. Disease-free survival (DFS) was defined as time from surgery to relapse or death due to any cause. Kaplan–Meier curves were used to graphically represent OS and DFS for study variables. The biomarker panel was then assessed with the log-rank test. Univariate and multivariate Cox regression models were also fitted for all variables, and crude hazard ratios (HRs) with 95% confidence intervals (CIs) were obtained for both outcomes.

The biomarker panel was constructed on the basis of variables that were statistically significant in previous analyses of OS and DFS. Univariate and multivariate Cox regression models were then fitted to the biomarker panel, including known risk factors, such as age and TNM stage, as covariates. Adjusted HRs were obtained for the biomarker panel, and the likelihood ratio test was used to assess its contribution to the model. Lastly, Harrell’s C index (crude and adjusted) for survival models was estimated to assess the predictive ability of the biomarker panel for OS and DFS. A value of 0.7 or higher was considered to indicate good discriminative ability.

All analyses were conducted using R, version 3.6.3.

## 3. Results

The final analysis included 144 patients (46 females and 98 males) with early-stage sporadic CC. The mean age at diagnosis was 72.2 years. A small majority of the cases (79, 54.9%) were right-sided tumors, most were of a high (≥50% gland forming) differentiation grade (118, 81.9%), with a similar split between stage II (55.6%) and stage III (44.4%) cases, and with an average of 6.7 resected lymph nodes at surgery. Eighteen patients (12.5% of the entire cohort) belonged to the CMS1 subgroup, 117 (81.2%) belonged to the CMS2/3 subgroup, and nine (6.2%) belonged to the CMS4 subgroup. Lymphatic vascular, blood vessel, and perineural invasion was found in 36 (25%), 42 (29.2%), and 32 (22.2%) patients, respectively. Baseline characteristics are presented in Table 1.

The incidence of dMMR was 12.5%. PD-L1 low expression was found in 80 patients (55.6%), GLUT-1 high expression was found in 81 patients (68.1%), loss of expression of e-cadherin was found in 66 patients (45.8%), loss of expression of MUC2 was found in 123 patients (85.4%), and loss of expression of CDX2 was found in 16 patients (11.1%). Expression of PD-L1 and MUC2 and loss of expression of CDX2 were significantly more frequently found in dMMR tumors.

The incidence of immunohistochemical characteristics is presented in Table 2. The baseline characteristics by MMR subgroup are shown in Table S2.

### 3.1. Study Variable Assessment

dMMR patients had the best prognosis in this cohort [54]. dMMR was, therefore, considered as a standalone first biomarker to be included in the panel. PD-L1, GLUT-1, e-cadherin, MUC2, and CDX2 were subsequently assessed for the rest of the cohort.

**Figure 1:**
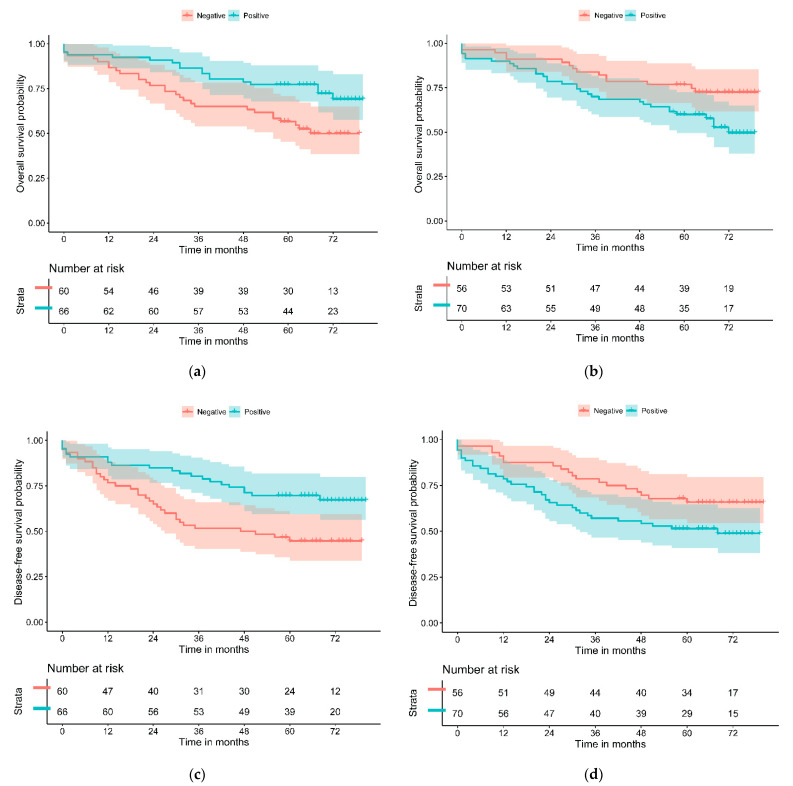
Kaplan–Meier curves of (**a**) PD-L1—L and (**b**) GLUT-1—H for overall survival and of (**c**) PD-L1—L and (**d**) GLUT-1—H for disease-free survival.

The relationship of OS and DFS with the study variables was assessed through univariate Cox regression and graphically represented with Kaplan–Meier curves and the log-rank test. PD-L1—L and GLUT-1—H significantly separated patients with the best and worst prognosis for both outcomes (Table 3 and Figure 1). PD-L1—L and GLUT-1—H resulted in a statistically significant difference in the univariate Cox regression model for OS (HR = 0.47, 95% CI (0.26–0.84), *p* = 0.010 and HR = 1.94, 95% CI (1.05–3.58), *p* = 0.029, respectively). Similar results were obtained for DFS. CDX2 was near statistical significance for DFS (HR = 2.15, 95% CI (0.92–5.04), *p* = 0.070). No significant differences were found for the other study variables.

Kaplan–Meier curves for all study variables are presented in Figure S2 for OS and Figure S3 for DFS.

In the univariate Cox regression analysis of baseline characteristics (Table S3), for OS, the risk of death rose by 7% with each additional year of age (HR = 1.07, 95% CI (1.03–1.1), *p* < 0.001) and was the only statistically significant baseline characteristic. The risk of relapse or death also significantly increased by 4% per additional year of age, while perineural invasion increased the risk of relapse or death significantly (HR = 1.88, 95% CI (1.05–3.34), *p* = 0.032). Consistent results were found for DFS. These variables were, therefore, considered in the multivariate analyses.

A multivariate Cox regression analysis was performed to assess for independence of variables found to be statistically significant in the previous analyses. The multivariate results were consistent with the univariate analyses. For OS, expression of PD-L1—L and GLUT-1—H was statistically significant and independent, conferring a 52% reduction in the risk of mortality (HR = 0.48, 95% CI (0.27–0.87), *p* = 0.016) and almost twice the risk of mortality (HR = 1.94, 95% CI (1.04–3.61), *p* = 0.036) respectively; similar results were found for DFS. Adding the loss of expression of CDX2 to the multivariate model showed it to have a tendency toward significance as increased risk of death (HR = 2.74, 95% CI (0.96–7.84), *p* = 0.061), and it was statistically significant for risk of death or relapse (HR = 2.77, 95% CI (1.06–7.23), *p* = 0.037).

### 3.2. Biomarker Panel and Risk Category Definition

Taking into account the above, MMR, PD-L1—L, and GLUT-1—H were considered in the biomarker panel construction. The risk categories were defined as low-risk when PD-L1—L = positive and GLUT-1—H = negative or dMMR, high-risk when PD-L1—L = negative and GLUT-1—H = positive, and medium-risk for any other combinations.

Despite the very small number of events, CDX2 showed a trend toward statistical significance for worse prognosis; thus, an exploratory sensitivity analysis was conducted. The loss of expression of CDX2 was added to the high-risk category of the biomarker panel, and the same analysis was performed as for the previous panel.

### 3.3. Assessing the Prognositic Capabilities of the Biomarker Panel

**Figure 2:**
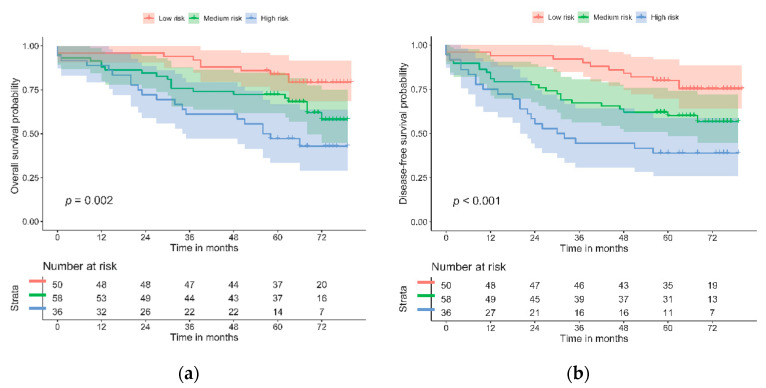
Kaplan–Meier curves for biomarker panel and risk categories: (**a**) OS; (**b**) DFS.

Regression models were derived, and survival was evaluated for the risk categories of the biomarker panel (Table 4 and Figure 2). The biomarker panel divided those patients with low, medium, and high risk of mortality and disease-free survival, independently of age, sex, TNM stage, and perineural invasion. According to the multivariate analysis, patients showing a lack of expression of PD-L1 and a high level of GLUT-1 expression have almost four times the risk of mortality (HR = 3.79, 95% CI (1.73–8.32), *p* = 0.001) of patients with the opposite types of expression or with dMMR. Similar results were found for DFS. These results were consistent with the survival analysis, as illustrated by the Kaplan–Meier curves. The multivariate model also showed that age and perineural invasion were independently associated with a significantly increased risk of mortality (*p* = 0.017).

The likelihood test was performed for the biomarker panel and its risk categories. It showed statistical significance in the univariate and multivariate Cox models for both OS (*p* = 0.002 and *p* = 0.002, respectively) and DFS (*p* = 0.001 and 0.003, respectively).

The goodness of fit for the risk scores was evaluated with Harrell’s C index. The scores from the multivariate analyses for OS and DFS were significant, with C values of 0.756 and 0.714, respectively.

Kaplan–Meier curves for the biomarker panel, by risk category, for OS and DFS both illustrated a statistically significant log-rank test (*p* = 0.002 and *p* = 0.001, respectively).

The exploratory sensitivity analysis that added CDX2 to the biomarker panel more clearly differentiated patients with higher risk and worse prognosis. Results from the multivariate analysis showed an increased risk of mortality or relapse (HR = 3.94, 95% CI (1.82–8.54), *p* < 0.001 for OS and HR = 3.63, 95% CI (1.78–7.41), *p* < 0.001 for DFS). The log-rank test and Kaplan–Meier curves were consistent with these results (Figure S4).

## 4. Discussion

In the present study, we found a selected biomarker panel in a cohort of early-stage CC patients to have significant prognostic value. dMMR, PD-L1, and GLUT-1 provided clearer predictions of the evolution of the disease in these patients. Expression of PD-L1 was significantly associated with longer survival and DFS, while GLUT-1 was significantly associated with poor OS and DFS. The biomarker panel was independent of sex, age, TNM stage, and perineural invasion. The final addition of patients with a loss of CDX2 expression to the high-risk category improved the numerical outcomes of the overall panel. This also helps clarify the outcomes for the few well-differentiated tumors that undergo this loss of expression and that have poor overall prognosis. To our knowledge, this is the first biomarker panel that successfully characterizes the prognosis of patients in early-stage colon cancer.

Colorectal cancer tumor biology and evolution vary greatly depending on various intrinsic and extrinsic factors, namely, type of tumor, localization, and stage [4,7]. To homogenize the population, we firstly focused on post curative-intent surgery stage II and III colorectal cancers. Secondly, formalin-fixed paraffin-embedded tissue samples from surgery had to be available for study. Thirdly, we excluded rectal cancers due to their different therapeutic approaches, etiology, biology, and prognosis [70,71,72,73,74]. Lastly, all patients had to be followed by the same treatment protocol established by the Colorectal Committee of our center.

Biomarkers were selected on the basis of their biological plausibility and the lessons learned through molecular characterization, especially by the CMS. In addition, it had to be possible to assess them by immunohistochemistry.

Arguably, the most important biomarker identified to date in CC is MSI/dMMR. It has a significant role as a predictive and prognostic tool for analyzing therapeutic response and clinical outcome. MSI is present in almost all proposed classifications, and it is currently assessed by MMR proteins through IHC in clinical practice and across tumor types. Assessing the expression of the MLH1, MSH2, MSH6, and PMS2 proteins, alongside BRAF, is currently helping to identify MSI or dMMR with clinical implications [4,7,8]. MSI is found mainly in the CMS1 subtype, since it is characterized by immune infiltration and activation [75]. Overall MSI convenes a good prognosis in the pre-metastatic stage, and its predictive capability has been reported along with targeted therapeutic options underway [76,77].

In our study, PD-L1 was expressed exclusively in immune cells found in either tumor-infiltrating cells or peritumoral area, where it provided a better prognosis for both OS and DFS. This is analogous to MSI tumors, which are enriched with CD3, CD4, CD20, and CD68 in the peritumoral region relative to MSS tumors, and which have also yielded a better prognosis [78]. Immune activation is the hallmark of CMS1; peritumoral immune cells can also be found in CMS3 although in lower quantities, along with expression of immune checkpoint inhibitors [75]. A previous study concluded that the prognostic capabilities of PD-L1, assessed by IHC in early-stage CC, make it possible to differentiate patients in the CMS2/3 subgroup [54].

When the high level of expression of GLUT-1 was assessed, it was mostly found in the membrane, and this indicated a poor prognosis. Metabolic dysregulation, particularly glucose metabolism dysregulation, is considered to be hallmark of the CMS3 subtype [13]. As previously suggested, the expression of GLUT-1 and its influence on prognosis may be a manifestation of tumor hypoxia. Consequently, the adaptive upregulation of aerobic glycolysis may be promoting tumor cell survival [79].

Although e-cadherin and MUC2 were not of prognostic value in our cohort, there seem to be some overlapping biological mechanisms, which may be worth further investigating. E-cadherin is a modulator of the WNT/β-catenin signaling pathway [34,38]. The WNT/β-catenin pathway is activated in most epithelial tumors, and it is consistently reported, particularly in the CMS2 subtype [80,81]. The pathway is mainly driven by APC mutations [82] and can also be linked to the activation of PD-L1 receptor [22]. The expression of MUC2 is regulated by epithelial growth factor (EGF) and TGF-α, and the activation signaling pathway goes through RAS kinases (KRAS, NRAS, and HRAS) [83]. CMS3 or metabolic type is characterized by the presence of KRAS and APC mutations, similarly to GLUT-1.

The expression of CDX2 progressively decreases with the transition from well to poorly differentiated cancer cell lines. This may be why the incidence reported in early-stage CRC is so low and difficult to assess. Regardless, our findings are in line with those of other publications [50,68,84]. As mentioned, the prognostic value of CDX2 is most often described in poorly differentiated tumors, but it is not so clear for well-differentiated or stage II and III tumors [68,85,86]. Pilati et al. reported that a lack of CDX2 expression in the CMS classification is useful for identifying poor-prognosis patients (CMS4/CDX2-loss), although, consistent with our study, CMS2 and CMS3 tumors rarely show loss of CDX2 [16].

As mentioned above, earlier efforts to implement a CMS classification in a clinical setting have failed, probably due to the robust methodology needed and the lack of prognostic or predictive capabilities, especially with CMS2 and CMS3, which at times comprise more than 50% of the population [87]. Additionally, in early-stage CC, the number of mesenchymal subtypes (CMS4) is very low, and they sometimes comprise groups with too few events to enable a meaningful statistical analysis [18,88].

These drawbacks are addressed by the proposed biomarker panel in this study. A literature review was performed to determine the adequate and simplest methodology for each biomarker. During the pathological assessment for this study, discrepancies were found in less than 5% of the samples. The main advantage of IHC may be the wide availability and hospital experience with the technique.

The results of this study suggest that patients should undergo conventional MSI/dMMR assessment in addition to analyzing two or three biomarkers by IHC. Using the specified antibodies in this study, any expression of PD-L1 (>1%), most of the tissue sample expressing GLUT-1 (>50%), and, if considered relevant, any loss of expression of CDX2 (<95%) should be assessed in the course of assembling the biomarker panel. In this way, it was possible to classify approximately one-third of the cohort (34.7%) as having a low-risk score and the best prognosis and around one-third of the population (29.9%) with a high-risk score and the worst prognosis. For the remaining third of patients with medium-risk scores (35.4%), other clinical variables, such as age, perineural invasion, or tolerance to chemotherapy, could be determining factors when deciding the most appropriate treatment intervention.

For the patients in the low-risk category, observation may be the current preference [4,7]. Several clinical trials are currently assessing the predictive value of immune checkpoint inhibitors in dMMR/MSI-H, high tumor mutational burden, or PD-L1-positive CRC patients in early-stage settings, some in phase III (NCT02912559, NCT03827044, NCT04304209, NCT04928807) and several more in phase II, given the encouraging results by Chalabi and colleagues [89]. Overall, either observation or low-level toxicity and personalized/targeted therapies may be preferable to full systemic chemotherapies for these patients.

For the patients in the high-risk category, more aggressive approaches may be considered to complement the upcoming immune checkpoint inhibitors. The addition of 5FU-based systems chemotherapies in combination with platins could prevent tumor progression early on in the process [4,7].

For the patients in the medium-risk category, additional variables may become more relevant when deciding the treatment approach. Other tumor characteristics, such as differentiated or undifferentiated histology, tumor budding, localization, vascular, perineural, or lymphatic invasion, and other patient characteristics, such as age, performance status, or tolerability to systemic treatments, might be the decisive factors for an early aggressive treatment.

The major limitations of this study are its retrospective and single-center nature. We acknowledge some others; even if patients were diagnosed and followed by the same oncology committee, there could be a lack of homogeneity among treatments during the patient journey, which could have influenced the study events. To diminish the impact of low-mortality events, DFS was assessed in parallel with OS, as it has shown to be a good indicator when mortality events are limited. However, the results obtained were consistent for the two outcomes. Since our cohort comprised mainly well-differentiated tumors, CDX2 events were rare and not enough to enable meaningful assessment. Few prognostic parameters have been acknowledged in the ESMO or NCCN guidelines; however, there is still a lack of robust evidence to support their value in all early-stage colorectal cancer patients, and they are limited to specific populations. These parameters were assessed in the present study, but the population in which they were assessed differs from our own, because, for the aforementioned reasons, we excluded patients with rectal cancer diagnosis and limited the cohort solely to patients with stage II and III colon cancer. Lastly, although specific antibodies may not be available in certain regions, and original clones may vary, a consistent assessment methodology should nevertheless be pursued.

Multicenter prospective studies could validate the proposed biomarker panel to clarify the prognosis of patients with early-stage CC. Additionally, larger cohorts could confirm its prognostic value and elucidate the effect and value of CDX2 as a complementary element to this biomarker panel.

## 5. Conclusions

In conclusion, this study provides a critical basis for the future development of an accessible IHC assessment capable of accurately describing the prognosis of patients with early-stage CC. The proposed biomarker panel may assist with personalization of treatment in the clinical setting and improve survival outcomes.

## Figures and Tables

**Table 1:** Baseline characteristics.

Characteristics	*N* (%) *n* = 144
Age * (years)	72.2 (9.6)
Range	48–93
Gender	
Female	46 (31.9)
Male	98 (68.1)
Localization	
Left	65 (45.1)
Right	79 (54.9)
Differentiation grade	
<50%	26 (18.1)
≥50%	118 (81.9)
Lymph node ratio	
Mean * (SD)	6.7 (12.1)
Histologic type	
Colloid	18 (12.5)
Adenocarcinoma NOS	125 (86.8)
Signet ring cell carcinoma	1 (0.7)
TNM Stage	
II	80 (55.6)
III	64 (44.4)
CMS	
CMS1	18 (12.5)
CMS2/3	117 (81.2)
CMS4	9 (6.2)
Lymphatic vascular invasion	
Negative	108 (75.0)
Positive	36 (25.0)
Blood vessel invasion	
Negative	102 (70.8)
Positive	42 (29.2)
Perineural invasion	
Negative	112 (77.8)
Positive	32 (22.2)

* Values are means. SD: standard deviation; CMS: consensus molecular subtype; NOS: not otherwise specified.

**Table 2:** Immunohistochemical characteristics.

Variable	*n* = 144 Total *N* (%)	*n* = 126 pMMR *N* (%)	*n* = 18 dMMR *N* (%)	*p*
PD-L1				
PD-L1—H (p/n)	29 (20.1)/115 (79.9)	19 (15.1)/107 (84.9)	10 (55.6)/8 (44.4)	<0.001 ^1^
PD-L1—L (p/n)	80 (55.6)/64 (44.4)	66 (52.4)/60 (47.6)	14 (77.8)/4 (22.2)	0.076 ^2^
GLUT-1				
GLUT-1—H (p/n)	81 (56.2)/63 (43.8)	70 (55.6)/56 (44.4)	11 (61.1)/7 (38.9)	0.849 ^2^
GLUT-1—L (p/n)	98 (68.1)/46 (31.9)	86 (68.3)/40 (31.7)	12 (66.7)/6 (33.3)	1.000 ^2^
e-Cadherin				
Positive	66 (45.8)	56 (44.4)	10 (55.6)	
Negative	78 (54.2)	70 (55.6)	8 (44.4)	0.527 ^2^
MUC2				
Positive	123 (85.4)	113 (89.7)	10 (55.6)	
Negative	21 (14.6)	13 (10.3)	8 (44.4)	0.001 ^1^
CDX2				
Positive	16 (11.1)	9 (7.1)	7 (38.9)	
Negative	128 (88.9)	117 (92.9)	11 (61.1)	0.001^1^

^1^ Fisher’s exact test; ^2^ chi-squared test; L: low, H: high; p/n: positive/negative; p/dMMR: proficient/deficient mismatch repair status.

**Table 3:** Univariate Cox regression for study variables.

Variable	Overall Survival HR	*p*	Disease-Free Survival HR	*p*
PD-L1				
PD-L1—H	0.45 (0.16–1.25)	0.088	0.46 (0.18–1.15)	0.090
PD-L1—L	0.47 (0.26–0.84)	0.010	0.47 (0.27–0.81)	0.007
GLUT-1				
GLUT-1—H	1.94 (1.05–3.58)	0.029	1.77 (1.01–3.09)	0.043
GLUT-1—L	1.62 (0.83–3.19)	0.143	1.48 (0.81–2.73)	0.201
e-Cadherin				
N/P	1.22 (0.69–2.16)	0.497	1.06 (0.62–1.81)	0.825
MUC2				
N/P	1.05 (0.41–2.65)	0.921	0.83 (0.33–2.09)	0.693
CDX2				
N/P	1.70 (0.67–4.31)	0.300	2.15 (0.92–5.04)	0.070

Reference; N: negative; P: positive; HR: hazard ratio.

**Table 4:** Regression models for risk classification in proposed biomarker panel.

Variable	Overall Survival HR		*p*	p LR *	Disease-Free Survival HR		*p*	p LR *
**Univariate Cox Model**								
Risk category								
Low	Ref.				Ref.			
Medium	2.10 (0.99–4.46)		0.054		2.07 (1.04–4.15)		0.039	
High	3.79 (1.77–8.11)		0.001	0.002	3.78 (1.86–7.66)		<0.001	0.001
**Multivariate Model**								
Age								
Years	1.08 (1.05–1.12)		<0.001		1.06 (1.03–1.09)		<0.001	
Sex								
Female	Ref.				Ref.			
Male	0.94 (0.51–1.73)		0.846		0.95 (0.54–1.67)		0.894	
Stage								
II	Ref.				Ref.			
III	1.04 (0.55–1.96)		0.906		1.14 (0.64–2.05)		0.652	
Perineural invasion								
No	Ref.				Ref.			
Yes	2.32 (1.16–4.62)		0.017		2.26 (1.19–4.31)		0.013	
Risk category								
Low	Ref.				Ref.			
Medium	1.70 (0.77–3.74)		0.186		1.61 (0.78–3.32)		0.198	
High	3.79 (1.73–8.32)		0.001	0.002	3.39 (1.64–7.02)		0.001	0.003
**Harrell’s C-index**	**C**	**D*xy***	**SD**		**C**	**D*xy***	**SD**	
Univariate model	0.637	0.275	0.071	<0.001	0.644	0.288	0.065	<0.001
Multivariate model	0.756	0.516	0.057	<0.001	0.714	0.428	0.060	<0.001

* LR: likelihood ratio test; Ref: reference; HR: hazard ratio.

## Data Availability

Full data for this study are available from the corresponding authors upon request.

## Supplementary Materials

The following are available online at https://www.mdpi.com/article/10.3390/cancers13235909/s1. Table S1: Summarized methodology for assessment and scoring; Table S2: Baseline characteristics by MMR subgroup; Table S3: Univariate Cox regression in baseline characteristics; Figure S1: Scanned images and example of scoring assessment; Figure S2: Kaplan–Meier curves for overall survival; Figure S3: Kaplan–Meier curves for disease-free survival; Figure S4: Kaplan–Meier curves for biomarker panel and risk categories including CDX2.
