# Supplementary material for: A Novel Prognostic Biomarker Panel for Early-Stage Colon Carcinoma
Source: Cancers (Basel). 2021 Nov 24;13(23):5909. doi: 10.3390/cancers13235909 (PMC8656725; doi:10.3390/cancers13235909)
Supplement: Supplementary file 1 [file cancers-13-05909-s001.zip › cancers-1419847 supplementary.pdf]

cancers

MDPI

Supplementary Materials: A Novel Prognostic Biomarker Panel for Early-Stage Colon Carcinoma

Table S1. Summarized methodology for assessment and scoring.

Antibody	Normal localization	Variation*	Control	Type of assessment	Scoring	Comments
MLH1<br>MSH2<br>MSH6<br>PMS2	Nuclear	Loss of expression	P: Normal colonic mucosa / Lymphocytes	Any staining	0: Present<br>1: Not present	MMR deficiency was defined as total loss of expression of any protein (MLH1, MSH2, MSH6 or PMS2) in the tumor
PD-L1	Membranous / Cytoplasmic	Overexpression	P: Amygdala	%[1]	0: <1%<br>1: 1% - <5%<br>2: 5% - <50%<br>3: ≥50%	At least 50 tumor cells viable for assessment. Low and High overexpression of PD-L1 was ascribed for scores of ≥1 and ≥2, respectively
GLUT1	Absence	Membranous / Cytoplasmic expression	P: Perineurium or erythrocytes<br>N: Normal colonic mucosa	%[2]	0: Absence<br>1: <30%<br>2: 30% - <50%<br>3: 50% - <90%<br>4: ≥90%	A score of 1 was mostly found in cytoplasm. Higher scores were mainly recorded in the membrane. Low and High expression was ascribed for scores of ≥2 and ≥3, respectively
E-Cadherin	Membranous	Loss of expression	P: Normal colonic mucosa<br>N: Stromal ovary tissue	%[2]	0: Absence<br>1: <10%<br>2: 10% - <50%<br>3: 50% - <80%<br>4: ≥80%	Loss of expression was concluded when % score multiplied by stain intensity was ≤6
				Stain intensity	0: Absence<br>1: Weak<br>2: Moderate<br>3: High	
MUC2	Cytoplasmic	Loss of expression	P: Normal colonic mucosa<br>N: Stromal ovary tissue	%[2]	0: <25%<br>1: 25% - <50%<br>2: ≥50%	Loss of expression was concluded for scores of 0 or 1
CDX2	Nuclear	Loss of expression	P: Normal colonic mucosa<br>N: Stromal ovary tissue	%[2]	0: <50%<br>1: 50% - <95%<br>2: ≥95%	Loss of expression was concluded for scores of 0 or 1

[1]Percentage area of positive immune cells within the total area of inflammation and tumor-related stroma of any intensity plus the percentage area of PD-L1 expressing tumor cells within the total tumor area of any intensity; [2]Percentage of immunostaining of tumor cell expression. MMR: Mis-match repair; P: Positive; N: Negative. *Variation expected from normality was considered as positive for statistical purposes.

Table S2. Baseline characteristics by MMR subgroup.

Variable	*n* =126<br>pMMR *N* (%)	*n* =18<br>dMMR *N* (%)	*p*
**Age**			
Mean (SD)	72.2 (9.5)	72.3 (10.6)	0.948[1]
Gender			
Female	40 (31.7)	6 (33.3)	
Male	86 (68.3)	12 (66.7)	1[2]
Localization			
Right	63 (50.0)	2 (11.1)	
Left	63 (50.0)	16 (88.9)	0.004[2]
Differentiation grade			
<50%	107(84.9)	11 (61.1)	
≥50%	19 (15.1)	7 (38.9)	0.022[3]
Lymph node ratio			
Mean* (SD)	6.9 (12.5)	5.2 (9.7)	0.436[4]
Histologic type			

Colloid	13 (10.3)	5 (27.8)	
Adenocarcinoma NOS	112(88.9)	13 (72.2)	0.053[3]
Signet ring cell carcinoma	1 (0.8)	0 (0.0)[δ]	
TNM Stage			
II	68 (54.0)	12 (66.7)	
III	58 (46.0)	6 (33.3)	0.447[2]
CMS			
CMS1	18 (100.0)	0 (0.0)	
CMS2/3	0 (0.0)	117(92.9)	<0.001[1]
CMS4	0 (0.0)	9 (7.1)	
Lymphatic vascular invasion			
Negative	92 (73.0)	16 (88.9)	
Positive	34 (27.0)	2 (11.1)	0.243[3]
Blood vessel invasion			
Negative	88 (69.8)	14 (77.8)	
Positive	38 (30.2)	4 (22.2)	0.678[2]
Perineural invasion			
Negative	96 (76.2)	16 (88.9)	
Positive	30 (23.8)	2 (11.1)	0.363[3]

[1]t-test; [2]chi-squared test; [3]Fisher's exact test; [4]Mann-Whitney U test; [δ]The single case of signet ring cell carcinoma has been excluded from the test. *SD: standard deviation; CMS: consensus molecular subtype; p/dMMR: proficient/deficient mismatch repair status; NOS: not otherwise specified.

Table S3. Univariate Cox regression in baseline characteristics.

**Variable**	**Overall survival HR**	***p***	**Disease-free survival HR**	***p***
**Age**				
Mean (SD)	1.07 [1.03 – 1.10]	<0.001	1.04 [1.01 – 1.07]	0.005
Gender				
Female* / Male	1.14 [0.61 – 2.13]	0.676	1.01 [0.57 – 1.79]	0.984
Localization				
Left* / Right	1.28 [0.72 – 2.28]	0.398	1.16 [0.68 – 1.99]	0.578
Differentiation grade				
<50%* / ≥50%	0.86 [0.38 – 1.91]	0.701	0.79 [0.36 – 1.75]	0.554
Lymph node ratio				
Mean* (SD)	1.01 [0.99 – 1.03]	0.231	1.01 [0.99 – 1.03]	0.446
Histologic type[δ]				
Colloid* /Adeno	0.73 [0.31 – 1.73]	0.498	0.77 [0.35 – 1.71]	0.534
TNM Stage				
II*/III	1.35 [0.76 – 2.40]	0.303	1.50 [0.88 – 2.57]	0.134
CMS				
CMS2/3* / CMS4	1.70 [0.67 – 4.31]	0.294	2.15 [0.92 – 5.04]	0.110
Lymphatic vascular invasion				
N*/P	0.81 [0.41 – 1.59]	0.531	0.91 [0.49 – 1.69]	0.760
Blood vessel invasion				
N*/P	1.46 [0.80 – 2.65]	0.223	1.68 [0.96 – 2.92]	0.074
Perineural invasion				
N*/P	1.73 [0.93 – 3.19]	0.093	1.88 [1.05 – 3.34]	0.041

*Reference; [δ] The single case of signet ring cell carcinoma has been excluded from the test. N: negative; P: positive; HR: hazard ratio; Adeno: adenocarcinoma; SD: standard deviation; CMS: consensus molecular subtype; p/dMMR: proficient/deficient mismatch repair status.

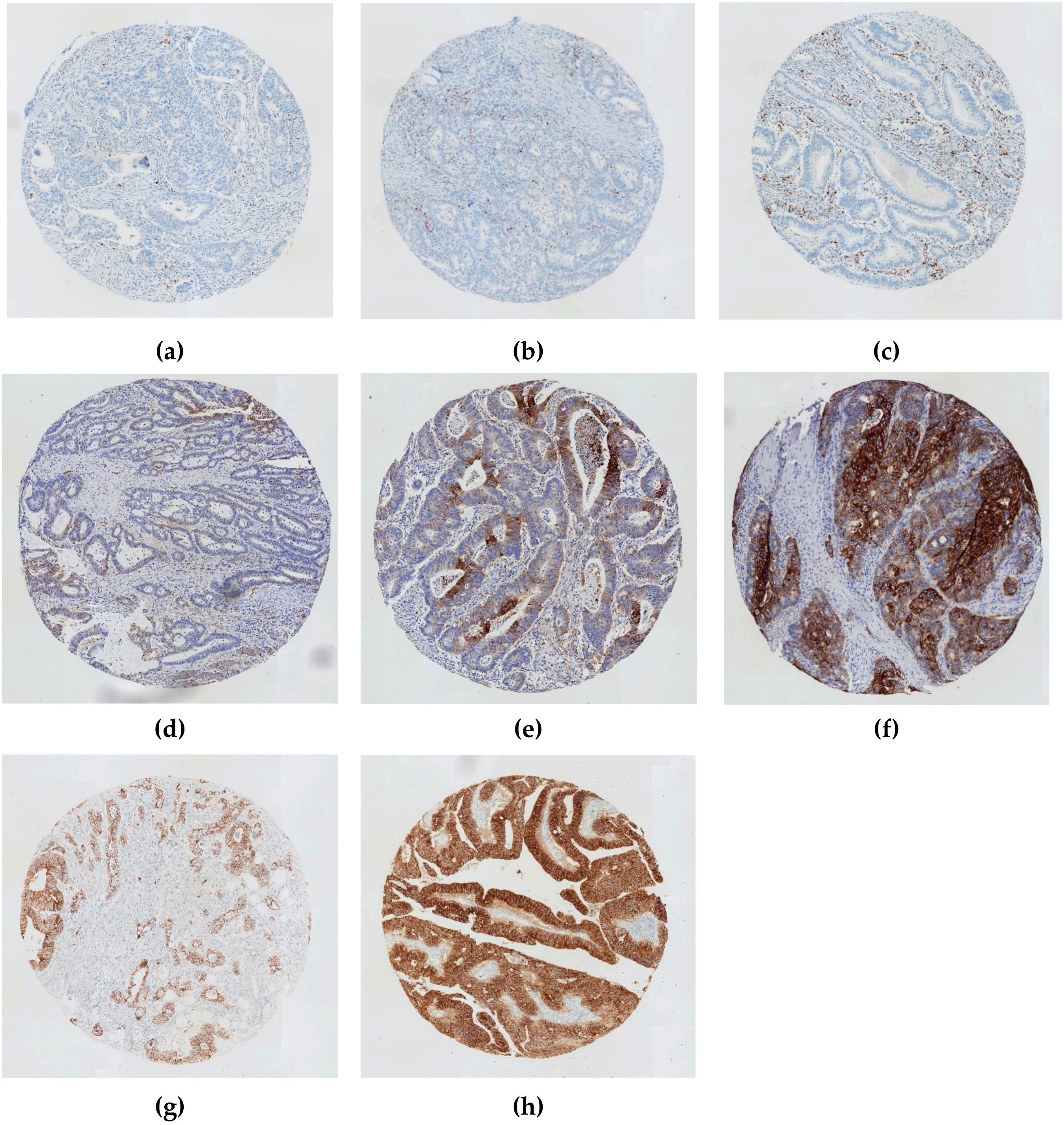

(a) (b) (c) (d) (e) (f) (g) (h)

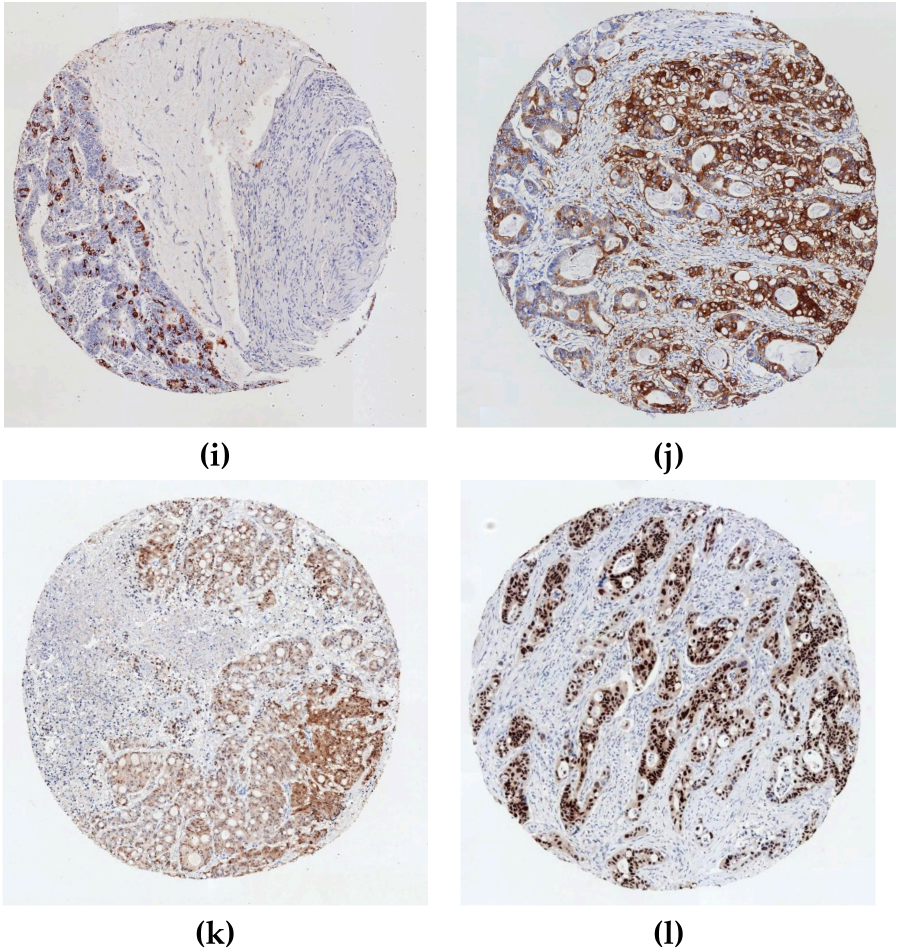

Figure S1. Scanned images and example of scoring assessment (core X4): (**a**) PD-L1 score 1; (**b**) PD-L1 score 2; (**c**) PD-L1 score 3; (**d**) GLUT-1 score 1; (**e**) GLUT-1 score 2; (**f**) GLUT-1 score 4; (**g**) e-Cadherin score ≤6; (**h**) e-Cadherin score >6; (**i**) MUC2 score 1; (**j**) MUC2 score 2; (**k**) CDX2 score 1; (**l**) CDX2 score 2.

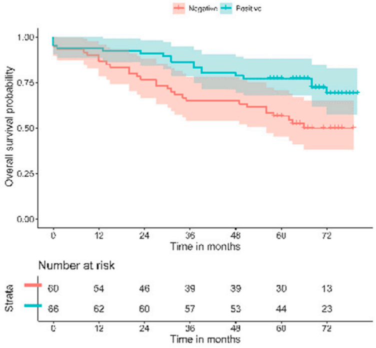
Negative Positive
Overall survival probability
1.00
0.75
0.50
0.25
0.00
0 12 24 36 48 60 72
Time in months
Number at risk
Strata
60 54 46 39 39 30 13
66 62 60 57 53 44 23

(**a**)

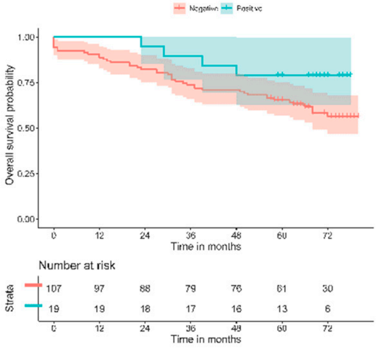
Negative Positive
Overall survival probability
1.00
0.75
0.50
0.25
0.00
0 12 24 36 48 60 72
Time in months
Number at risk
Strata
107 97 88 79 76 61 30
19 19 18 17 16 13 6

(**b**)

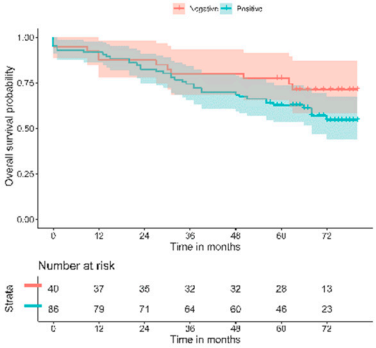
Negative Positive
Overall survival probability
1.00
0.75
0.50
0.25
0.00
0 12 24 36 48 60 72
Time in months
Number at risk
Strata
40 37 35 32 32 28 13
86 79 71 64 60 46 23

(**c**)

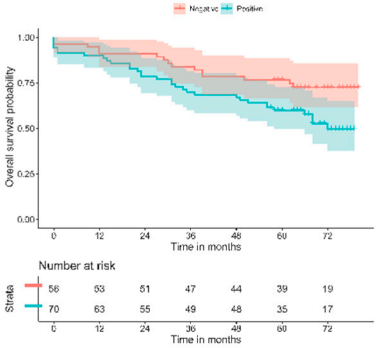
Negative Positive
Overall survival probability
1.00
0.75
0.50
0.25
0.00
0 12 24 36 48 60 72
Time in months
Number at risk
Strata
56 53 51 47 44 39 19
70 63 55 49 48 35 17

(**d**)

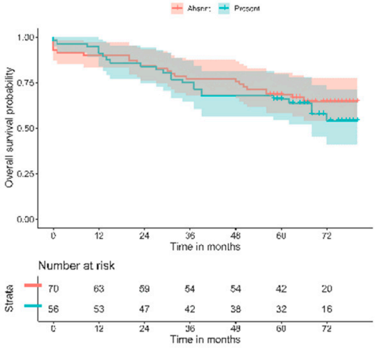
Absent Present
Overall survival probability
1.00
0.75
0.50
0.25
0.00
0 12 24 36 48 60 72
Time in months
Number at risk
Strata
70 63 59 54 54 42 20
56 53 47 42 38 32 16

(**e**)

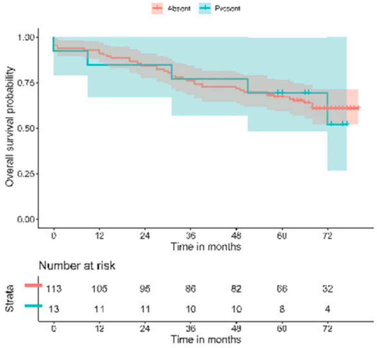
Absent Present
Overall survival probability
1.00
0.75
0.50
0.25
0.00
0 12 24 36 48 60 72
Time in months
Number at risk
Strata
113 105 95 86 82 66 32
13 11 11 10 10 8 4

(**f**)

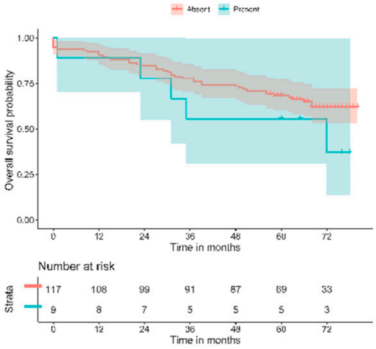
Absent Present
Overall survival probability
1.00
0.75
0.50
0.25
0.00
0 12 24 36 48 60 72
Time in months
Number at risk
Strata
117 108 99 91 87 69 33
9 8 7 5 5 5 3

(**g**)

Figure S2. Kaplan–Meier curves for overall survival: (**a**) PD-L1 – L; (**b**) PD-L1 – H; (**c**) GLUT1 – L; (**d**) GLUT-1 – H; (**e**) e-Cadherin; (**f**) MUC2; (**g**) CDX2.

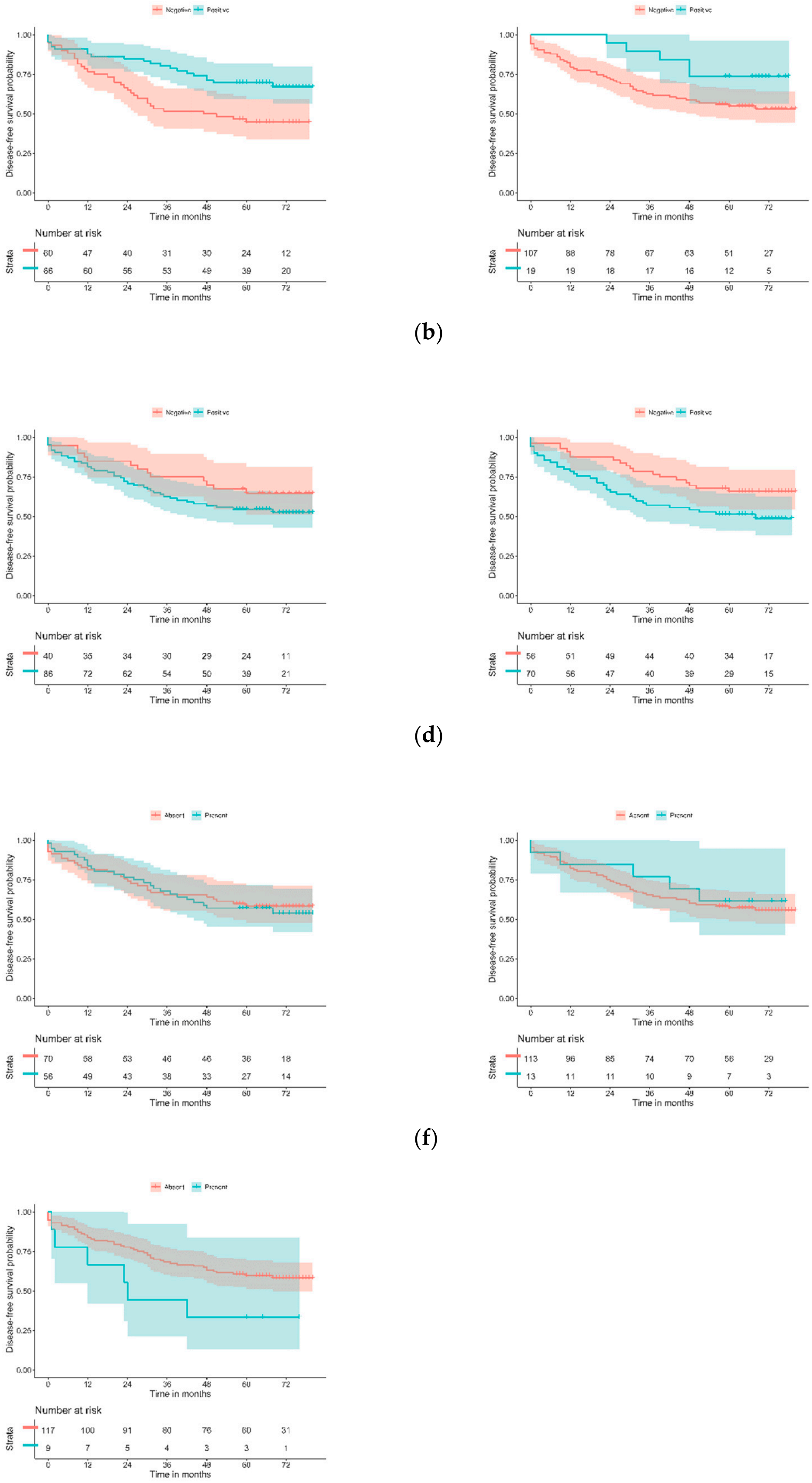
Negative
Positive
Absent
Present
Disease-free survival probability
Time in months
Number at risk
Strata
80 47 40 31 30 24 12
66 60 56 53 49 39 20
107 88 78 67 63 51 27
19 19 18 17 16 12 5
40 36 34 30 29 24 11
86 72 62 54 50 39 21
56 51 49 44 40 34 17
70 56 47 40 39 29 15
70 58 53 46 46 36 18
56 49 43 38 33 27 14
113 96 85 74 70 56 29
13 11 11 10 9 7 3
117 100 91 80 76 60 31
9 7 5 4 3 3 1

(**a**) (**b**) (**c**) (**d**) (**e**) (**f**) (**g**)

Figure S3. Kaplan–Meier curves for disease-free survival: (**a**) PD-L1 – L; (**b**) PD-L1 – H; (**c**) GLUT1 – L; (**d**) GLUT-1 – H; (**e**) e-Cadherin; (**f**) MUC2; (**g**) CDX2

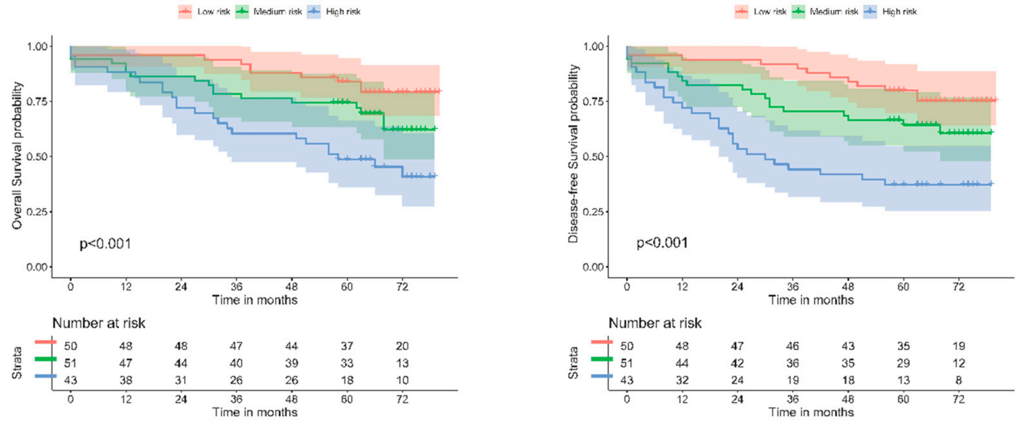

(**a**) (**b**)

Figure S4. Kaplan–Meier curves for biomarker panel and risk categories including CDX2: (**a**) OS; (**b**) DFS.
