# Supplementary figures and images for: A Novel Prognostic Biomarker Panel for Early-Stage Colon Carcinoma
Source: Cancers (Basel). 2021 Nov 24;13(23):5909. doi: 10.3390/cancers13235909 (PMC8656725; doi:10.3390/cancers13235909)

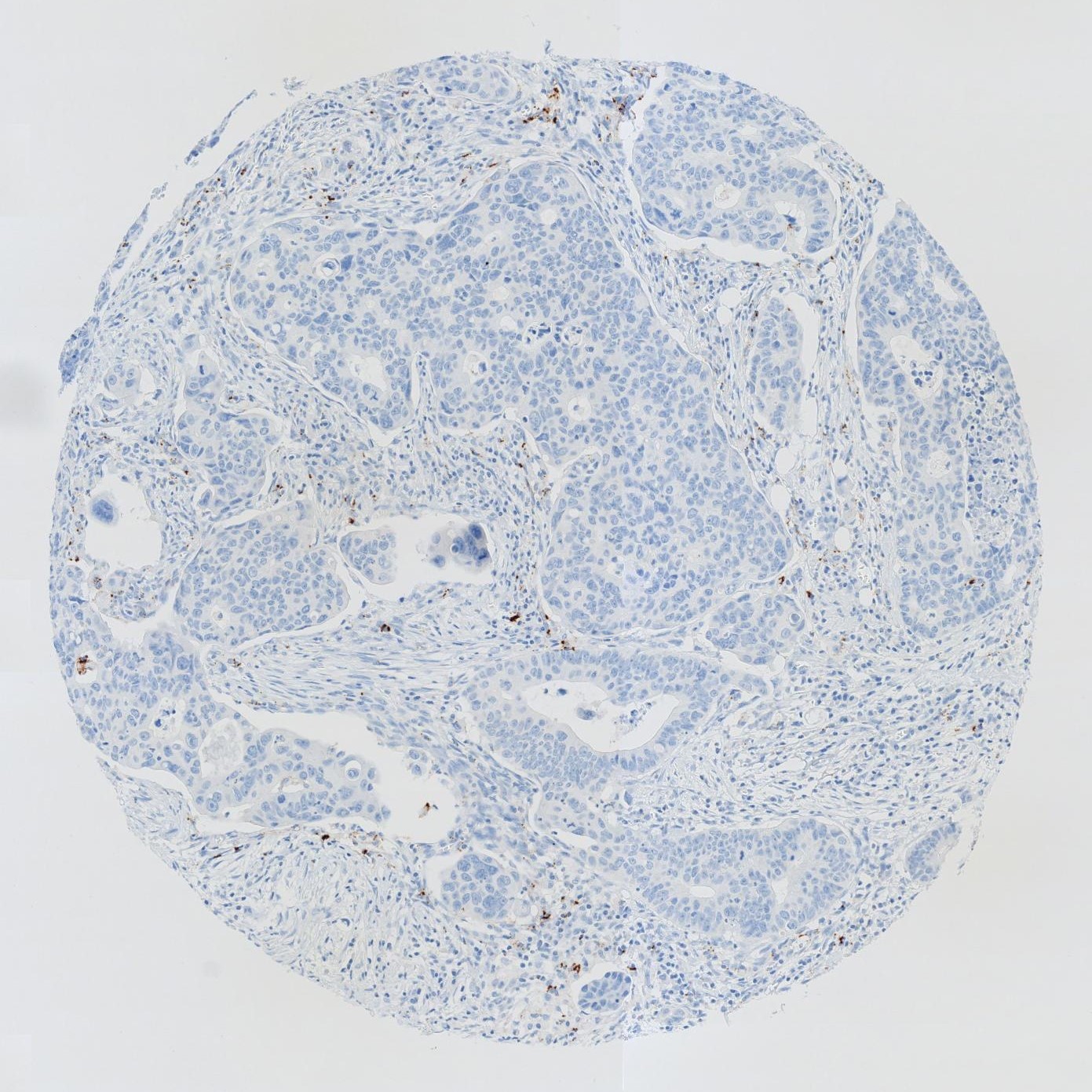

Supplement: Supplementary file 1 [file cancers-13-05909-s001.zip › Supplementary Original Figures/Supplementary Figure S1a.jpg]

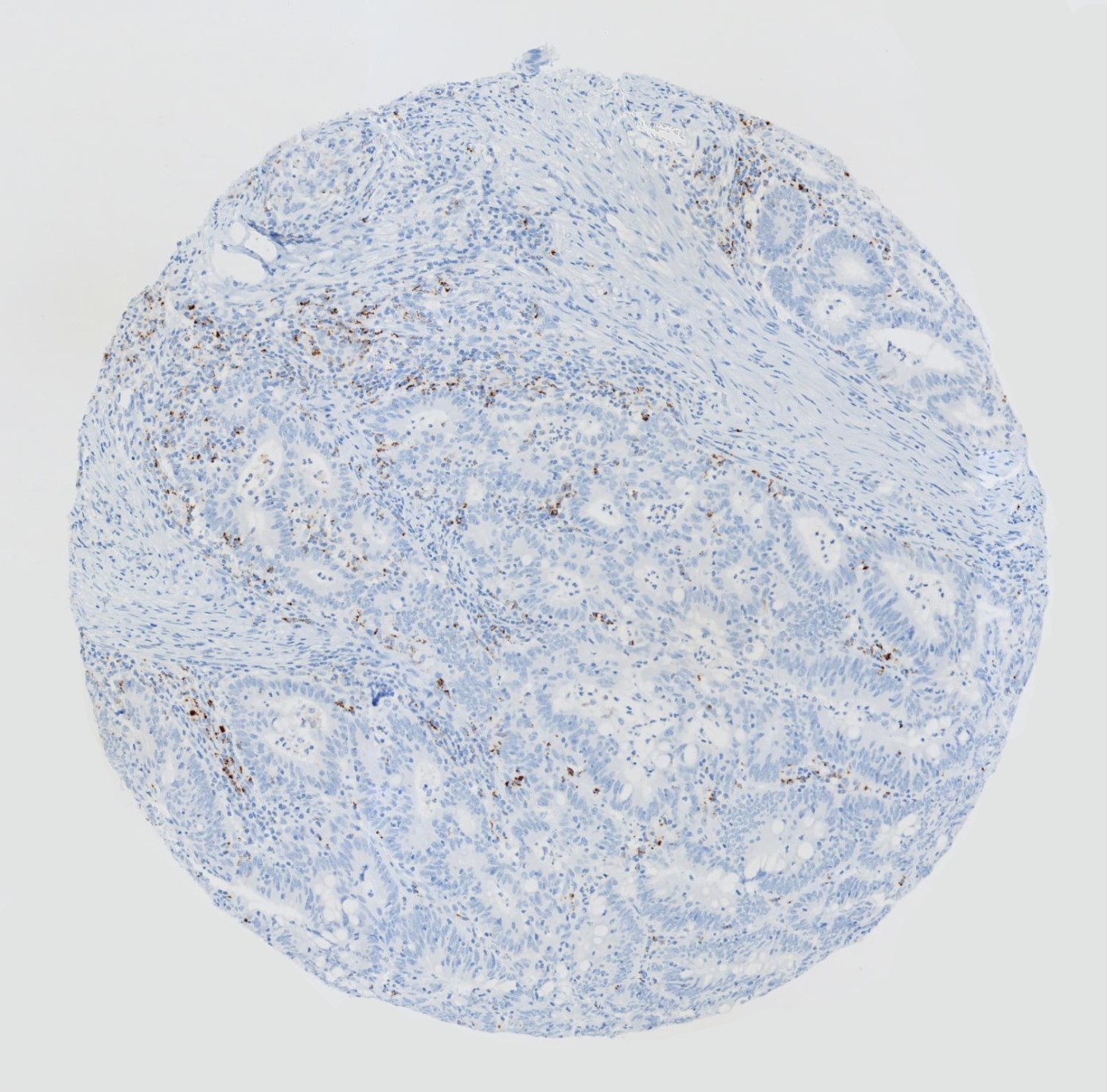

Supplement: Supplementary file 1 [file cancers-13-05909-s001.zip › Supplementary Original Figures/Supplementary Figure S1b.jpg]

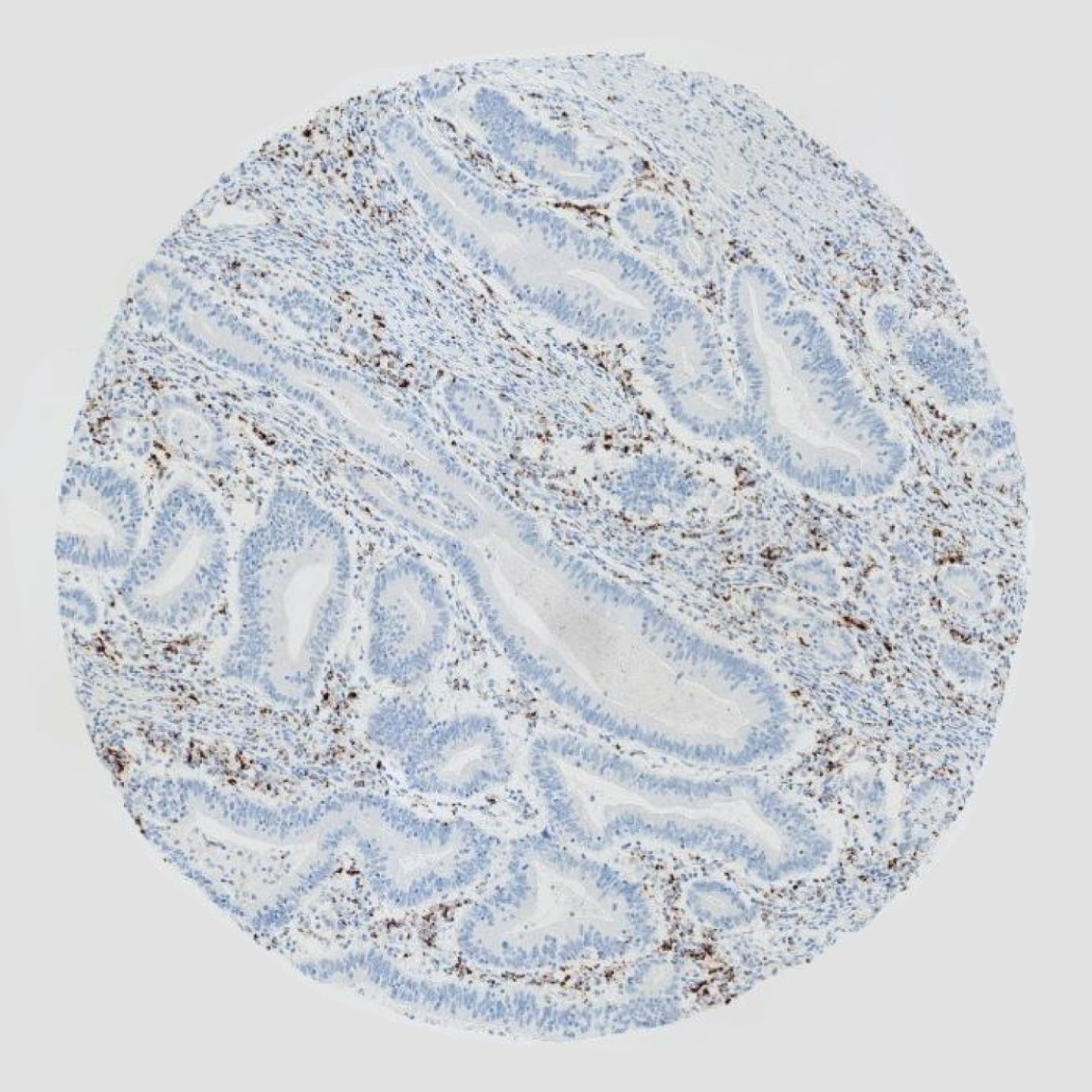

Supplement: Supplementary file 1 [file cancers-13-05909-s001.zip › Supplementary Original Figures/Supplementary Figure S1c.jpg]

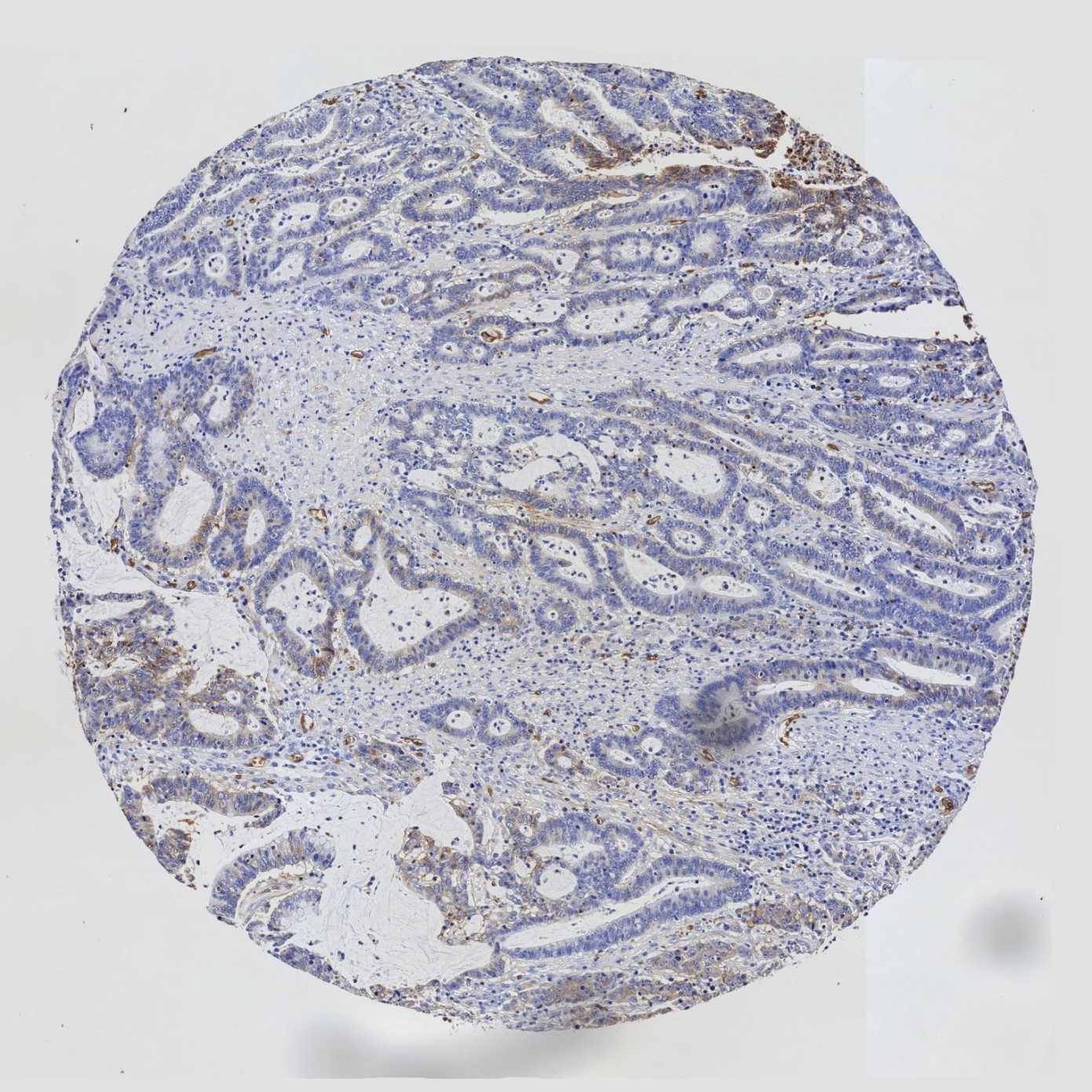

Supplement: Supplementary file 1 [file cancers-13-05909-s001.zip › Supplementary Original Figures/Supplementary Figure S1d.jpg]

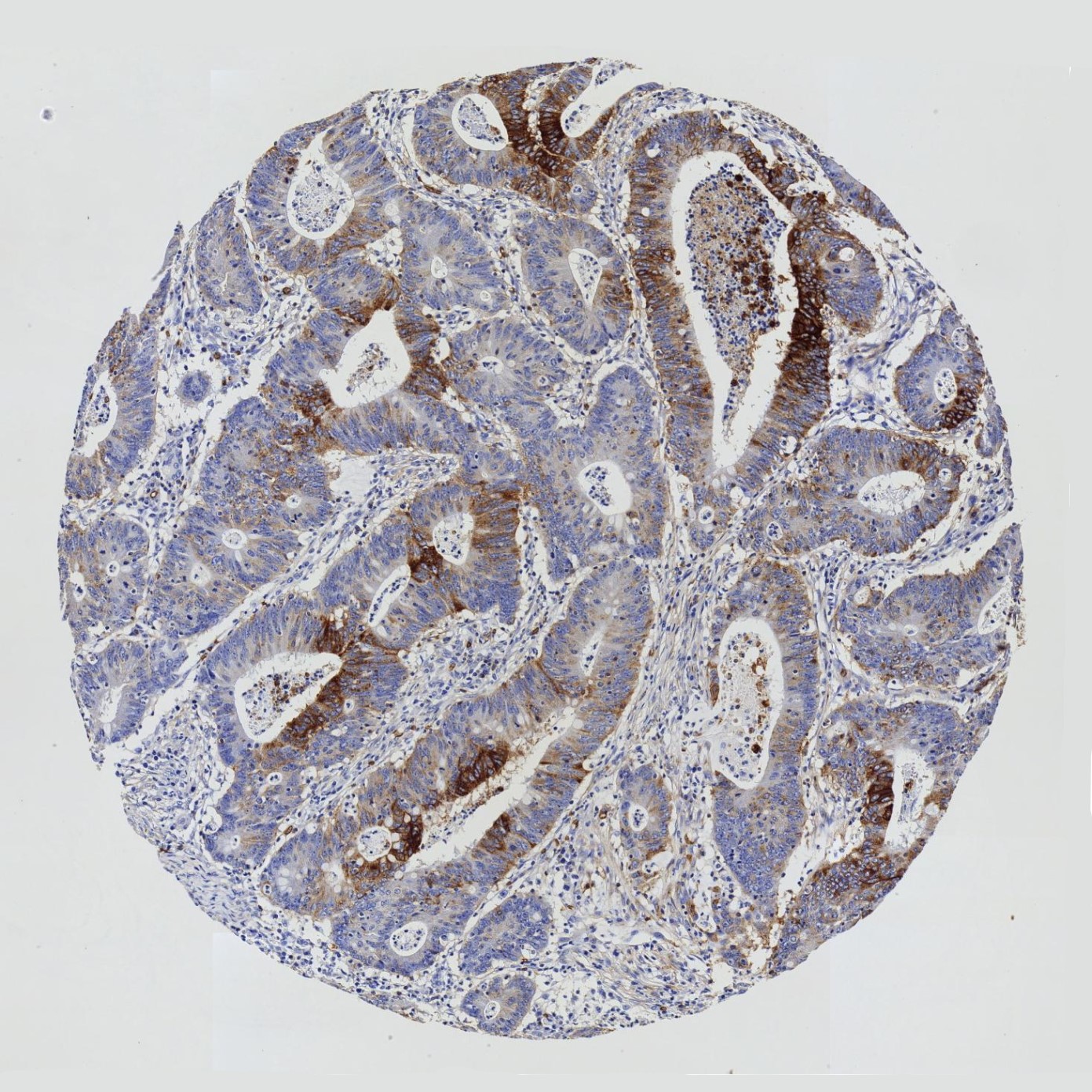

Supplement: Supplementary file 1 [file cancers-13-05909-s001.zip › Supplementary Original Figures/Supplementary Figure S1e.jpg]

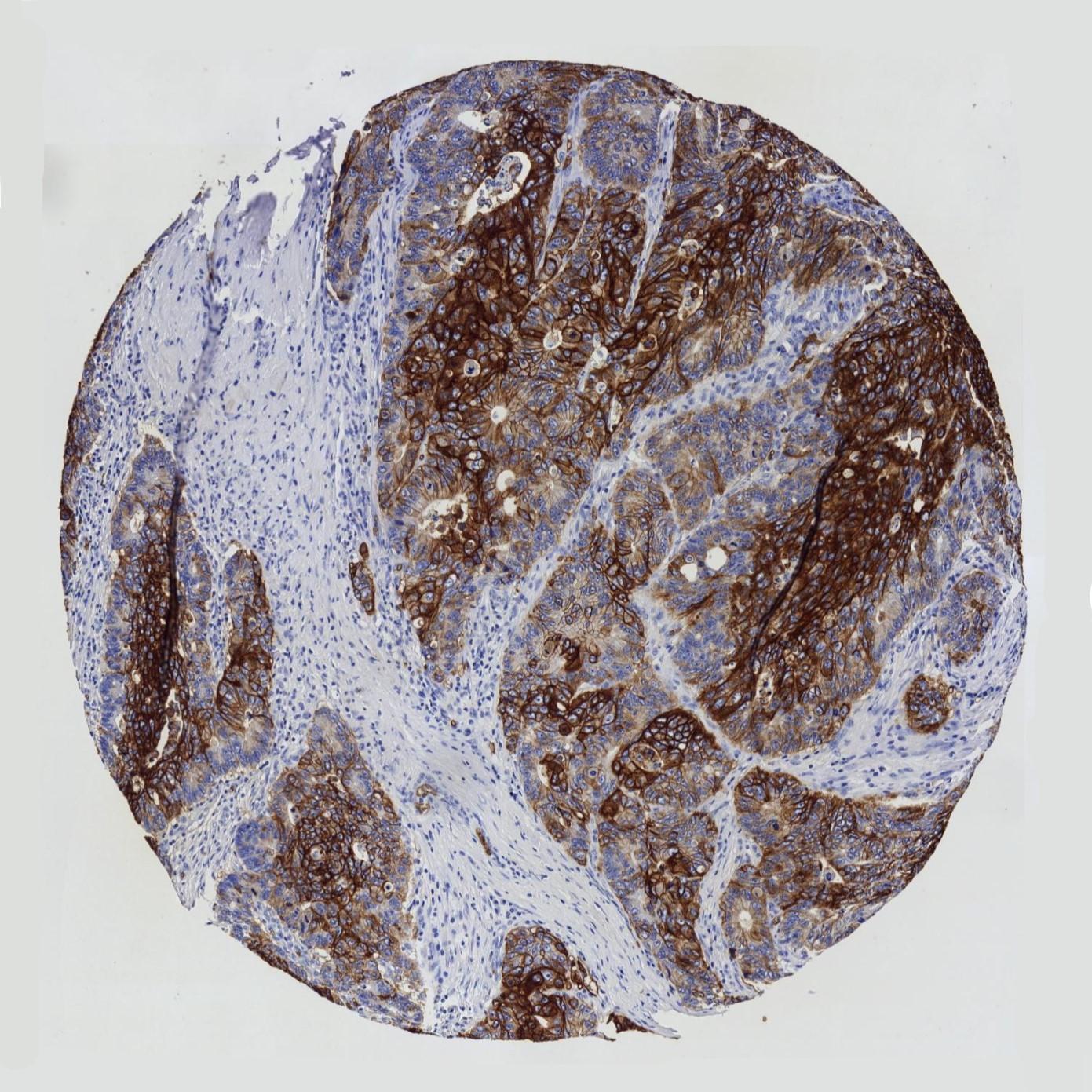

Supplement: Supplementary file 1 [file cancers-13-05909-s001.zip › Supplementary Original Figures/Supplementary Figure S1f.jpg]

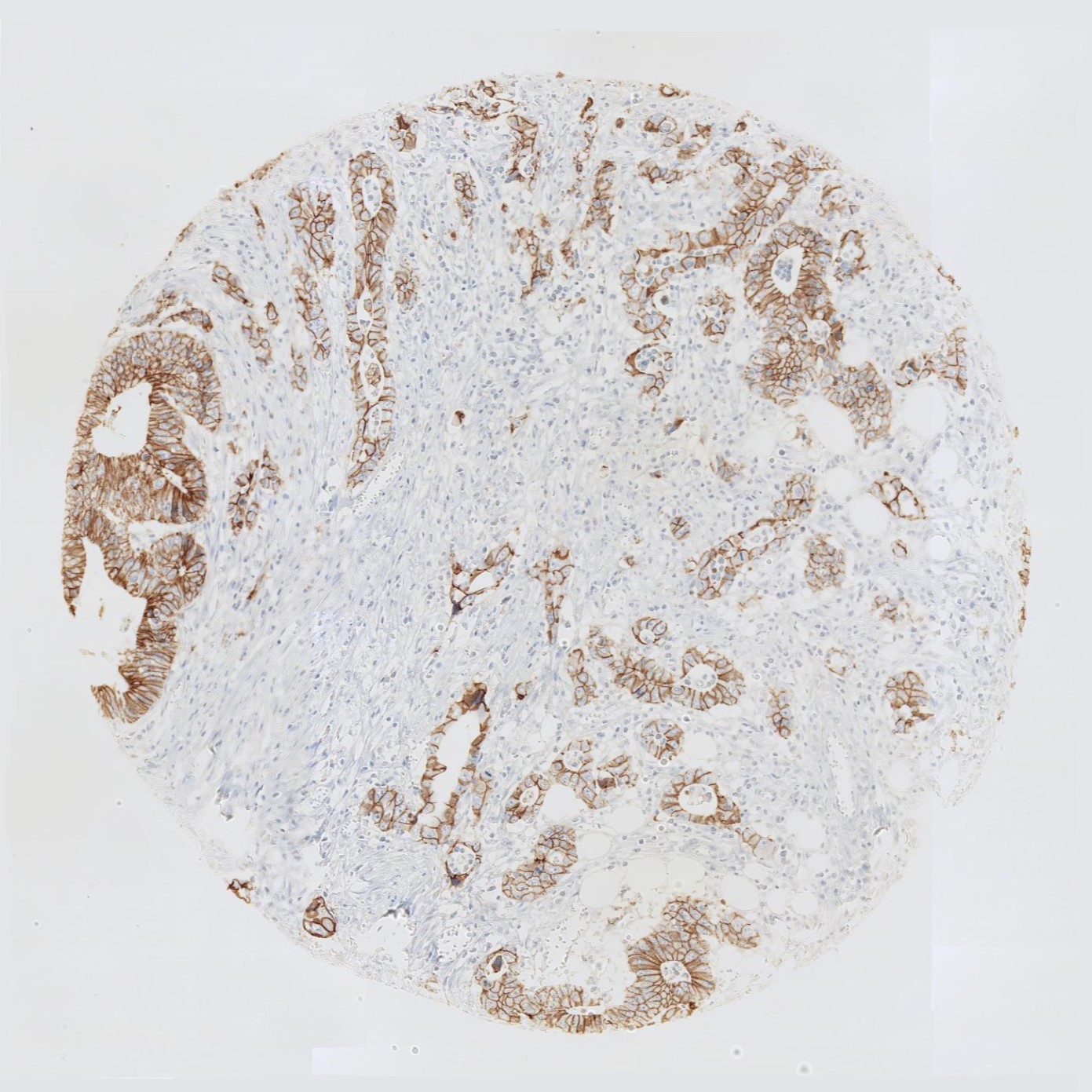

Supplement: Supplementary file 1 [file cancers-13-05909-s001.zip › Supplementary Original Figures/Supplementary Figure S1g.jpg]

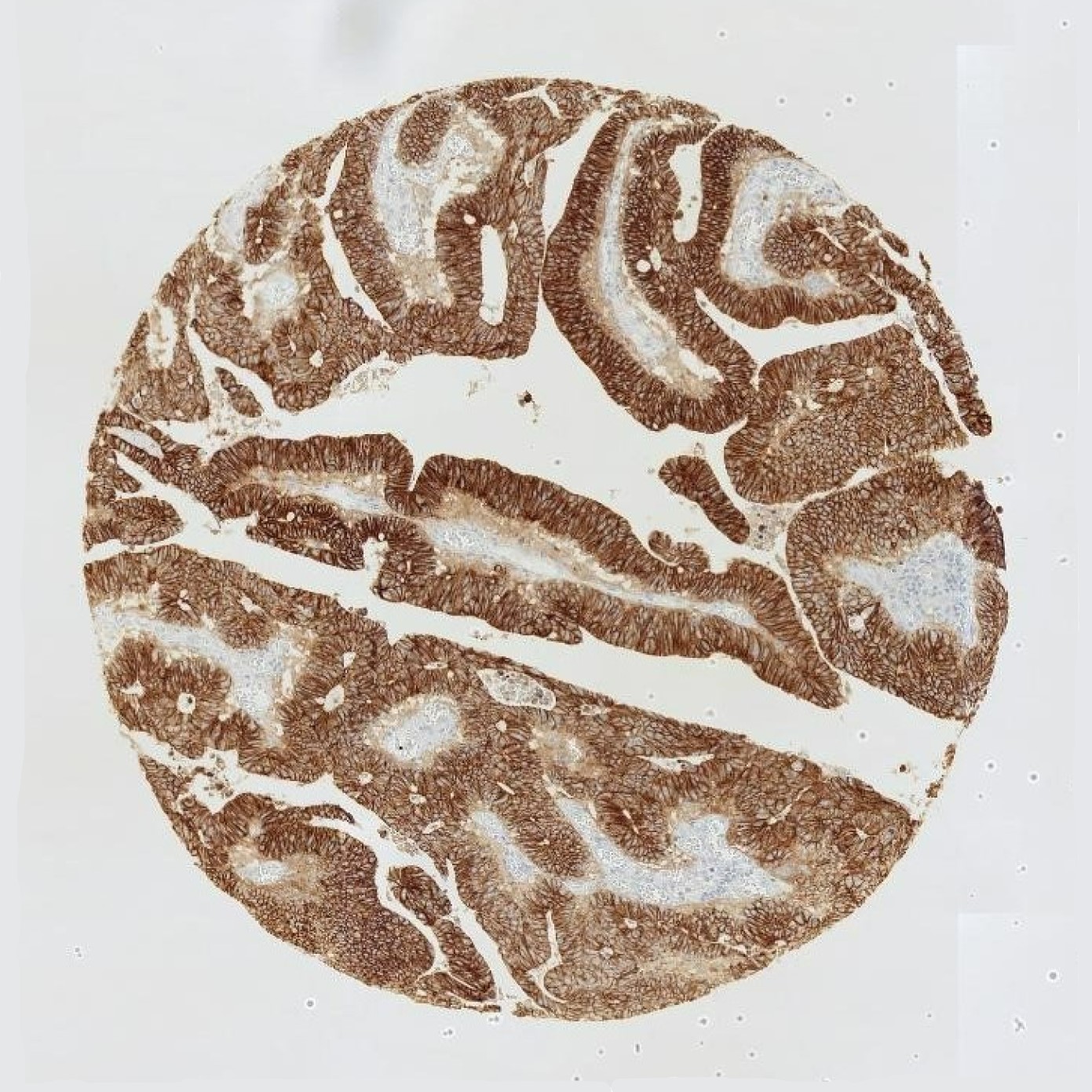

Supplement: Supplementary file 1 [file cancers-13-05909-s001.zip › Supplementary Original Figures/Supplementary Figure S1h.jpg]

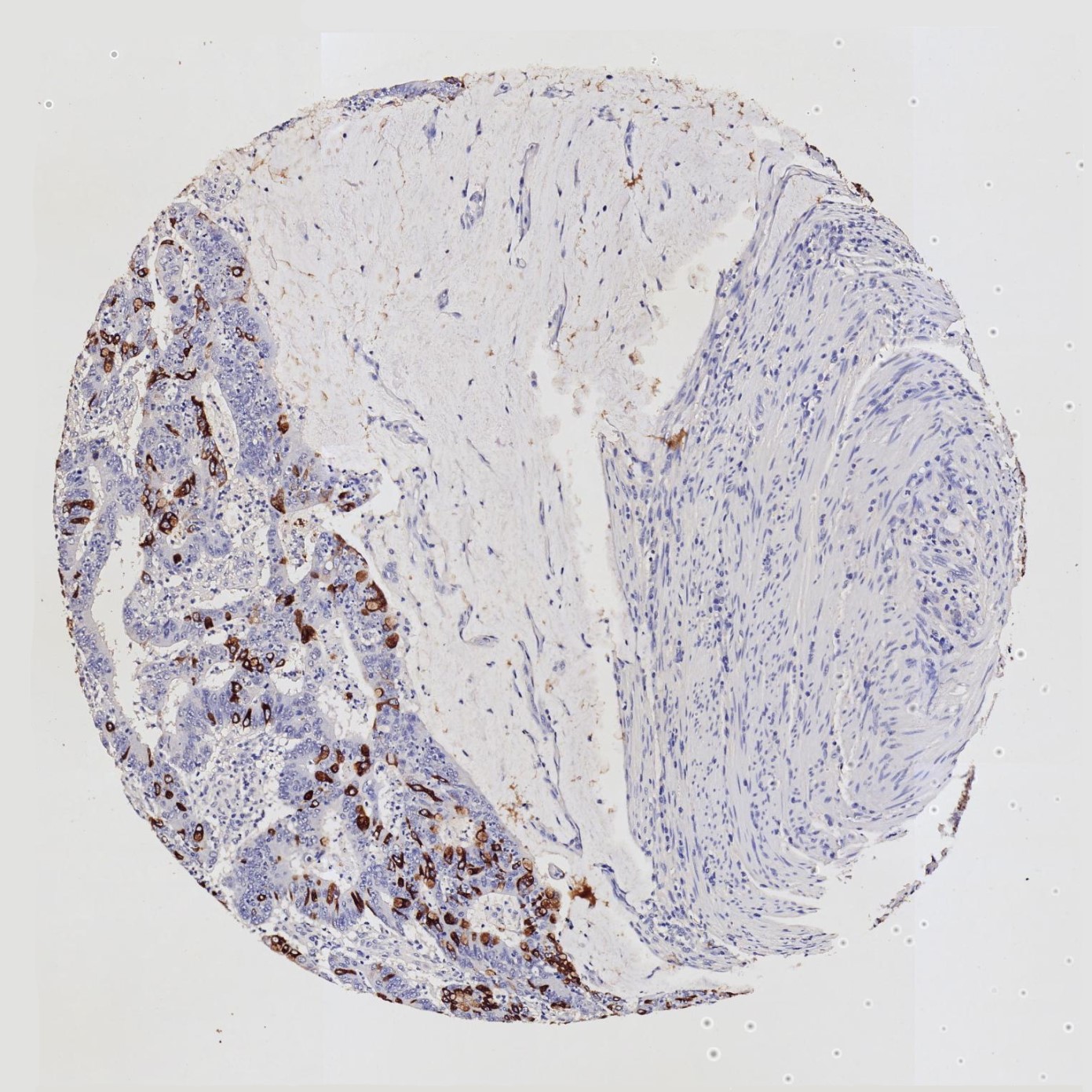

Supplement: Supplementary file 1 [file cancers-13-05909-s001.zip › Supplementary Original Figures/Supplementary Figure S1i.jpg]

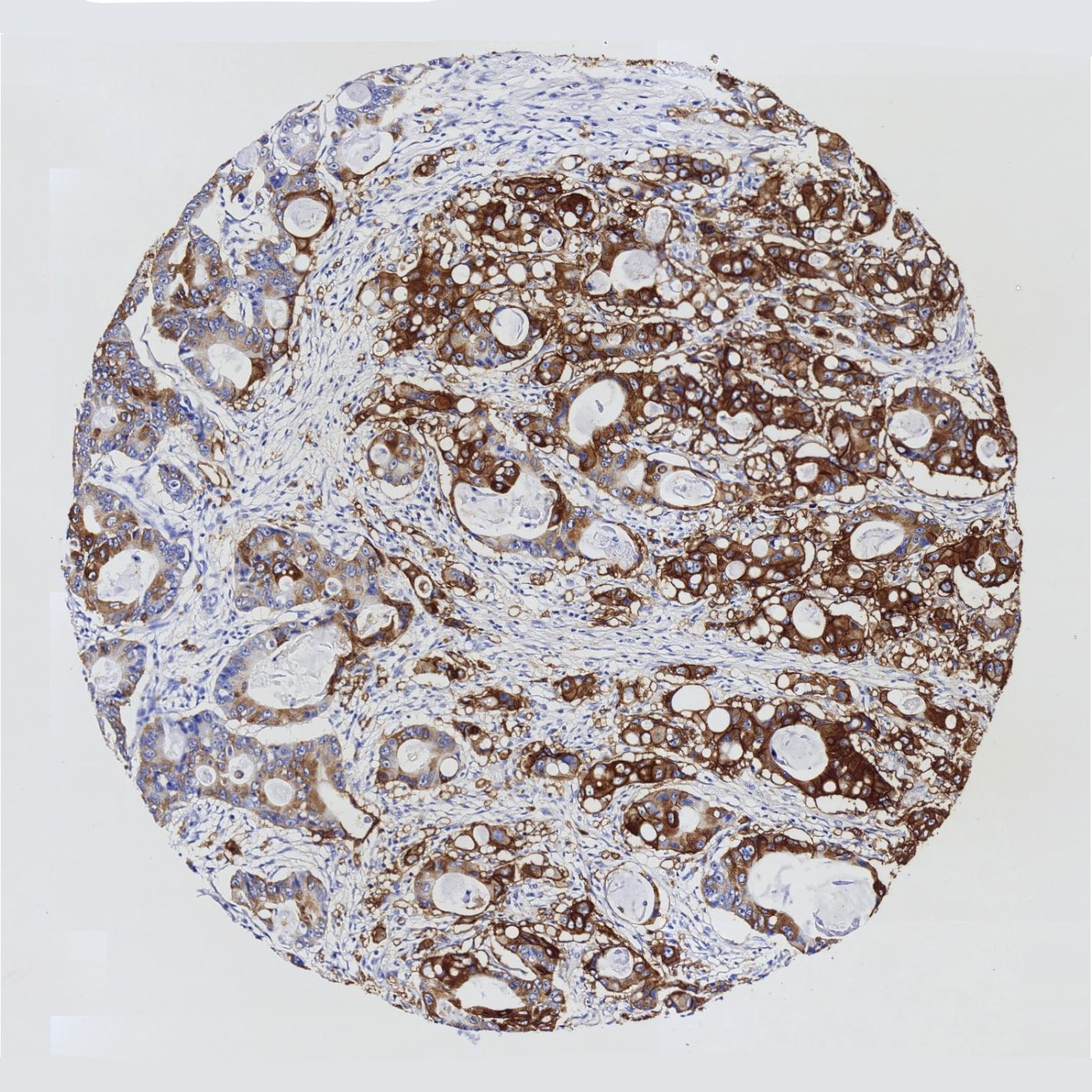

Supplement: Supplementary file 1 [file cancers-13-05909-s001.zip › Supplementary Original Figures/Supplementary Figure S1j.jpg]

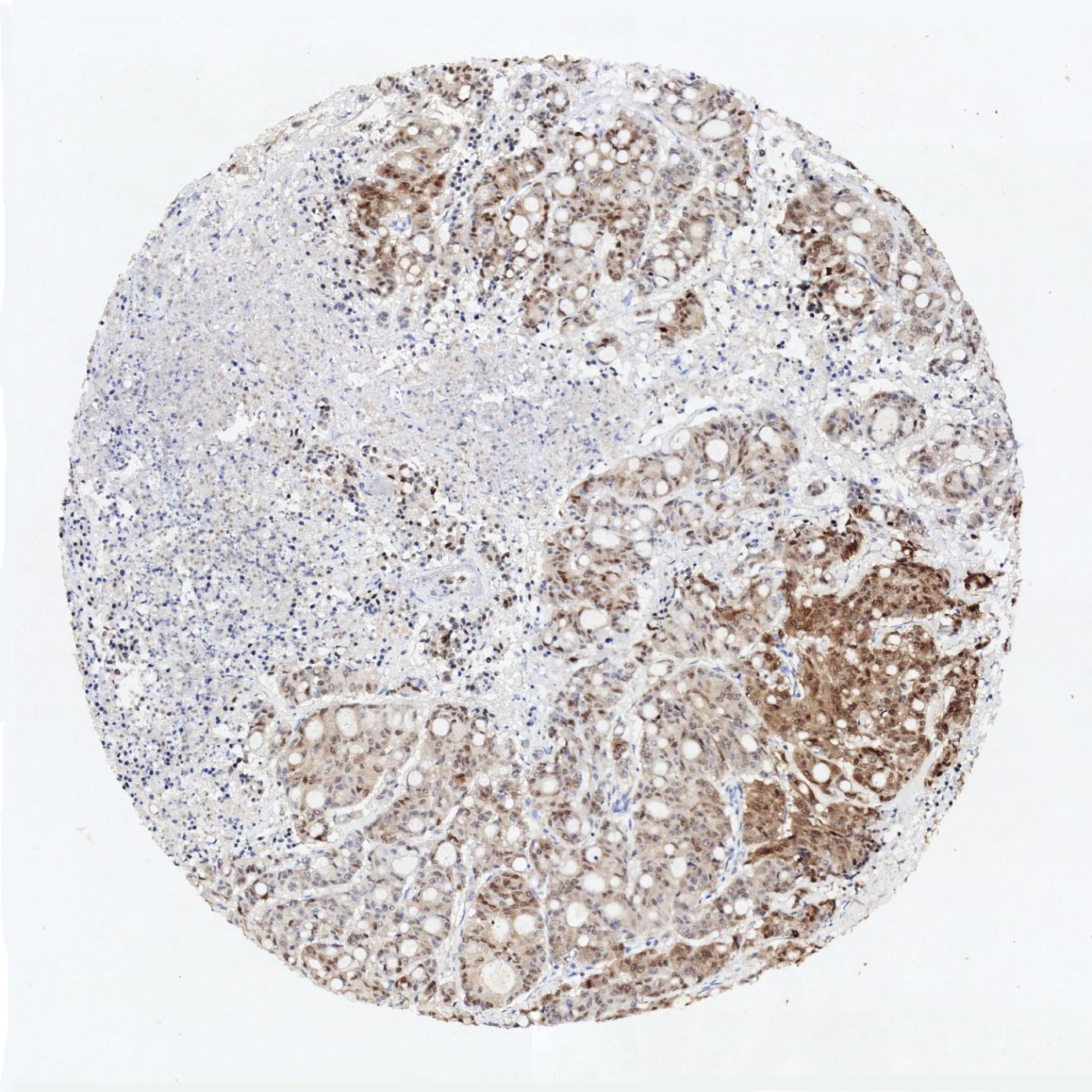

Supplement: Supplementary file 1 [file cancers-13-05909-s001.zip › Supplementary Original Figures/Supplementary Figure S1k.jpg]

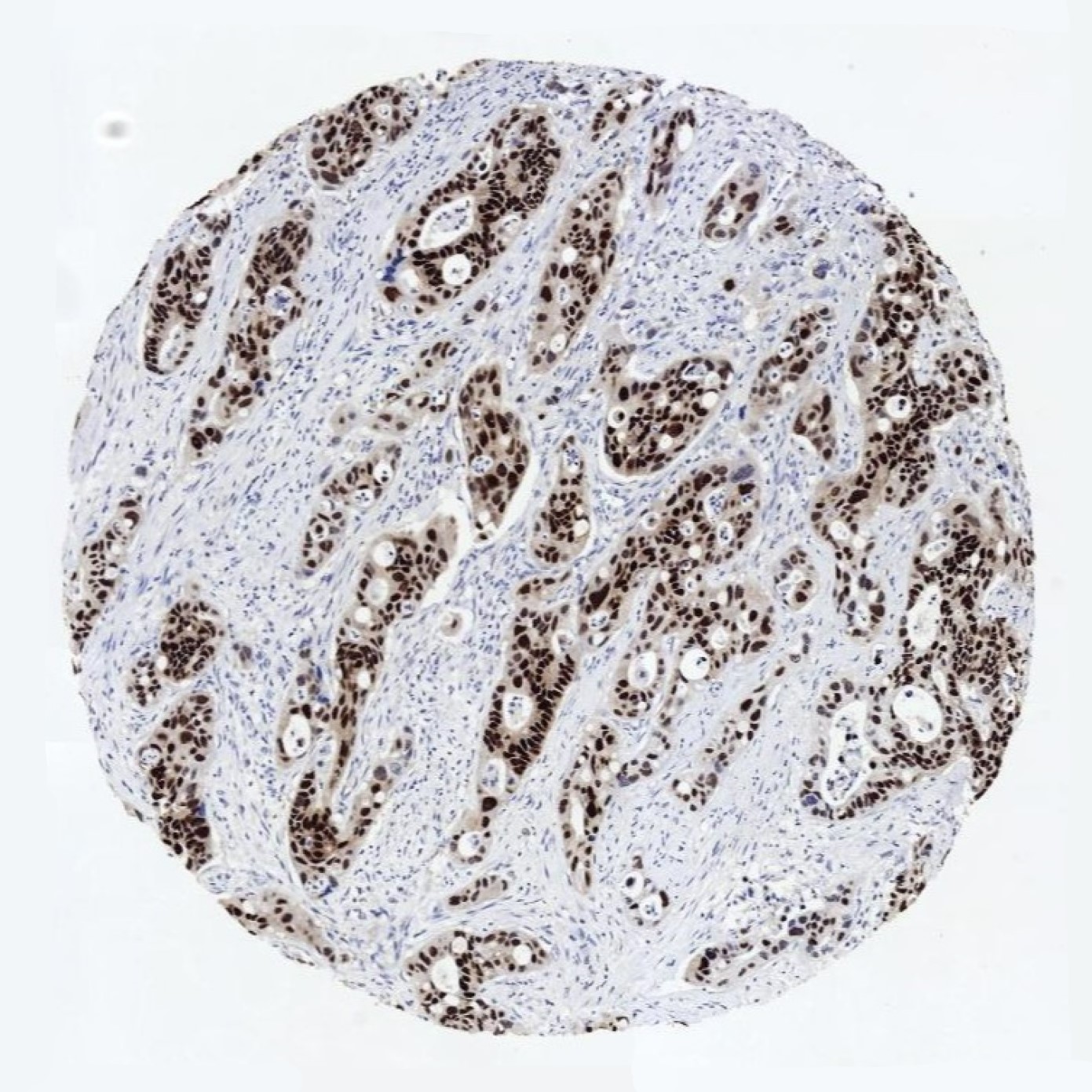

Supplement: Supplementary file 1 [file cancers-13-05909-s001.zip › Supplementary Original Figures/Supplementary Figure S1l.jpg]
